# Porous carbons: a class of nanomaterials for efficient adsorption-based hydrogen storage

**DOI:** 10.1039/d4lf00215f

**Published:** 2024-10-30

**Authors:** Lila A. M. Mahmoud, Jemma L. Rowlandson, David J. Fermin, Valeska P. Ting, Sanjit Nayak

**Affiliations:** a School of Chemistry, Cantocks Close, University of Bristol Bristol BS8 1TS UK; b Bristol Composites Institute, University of Bristol Bristol BS8 1TR UK; c School of Electrical, Electronic and Mechanical Engineering, University of Bristol Bristol BS8 1TR UK; d School of Civil, Aerospace and Design Engineering, University of Bristol Bristol BS8 1TR UK s.nayak@bristol.ac.uk; e Research School of Chemistry, Australian National University Canberra ACT 2601 Australia valeska.ting@anu.edu.au

## Abstract

Hydrogen has become a promising clean energy source as governments worldwide aim to reduce their reliance on fossil fuels and achieve net-zero emissions. However, a major barrier for hydrogen economy is the challenges associated with the efficient storage of hydrogen, due to its low density, ultra-low boiling point, and extreme volatility. Current practice of using high-pressure tanks has safety concerns and is costly. As a potential solution, adsorption-based hydrogen storage using porous materials has shown great promise due to fast kinetics and their ability to store a comparable amount of hydrogen at much lower pressure. This approach takes advantage of physisorption of hydrogen in porous materials with high surface areas. A number of different classes of materials have been studied for adsorption-based hydrogen storage. Among these materials, porous carbon has shown great promise due to its high surface area, tunable pore size, versatile surface chemistry, scalability, and high chemical and thermal stability. This review provides a comprehensive overview of porous carbon materials, such as graphene, carbon nanotubes, and activated carbons, for hydrogen storage. We delve into the fundamental principles and mechanisms behind adsorptive hydrogen storage, focusing on the critical roles of surface area, pore size, and surface chemistry in determining hydrogen uptake. Strategies to enhance hydrogen storage capacity through structural and chemical modifications are discussed. Additionally, we examine the life cycle assessment of porous carbons and explore recent advancements in machine learning applications to optimize their performance. Finally, we offer insights into the future outlook of porous carbons as a sustainable hydrogen storage solution.

## Abstract 2

This review provides an overview of porous carbon materials and their hydrogen storage properties with insight on strategic improvement by structural and chemical modifications and current trends of using machine learning and lifecycle assessment.

## Graphical abstract

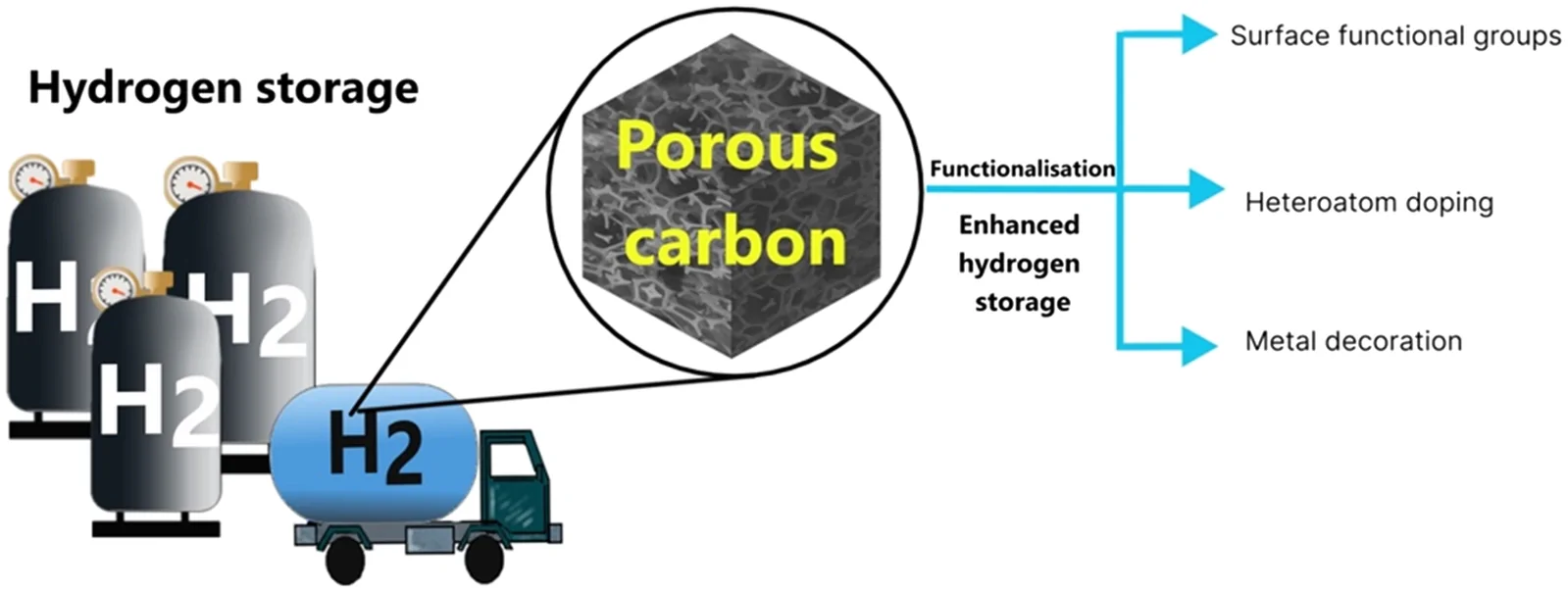

## 1. Hydrogen as a sustainable energy source: challenges and opportunities

**Scheme 1:**
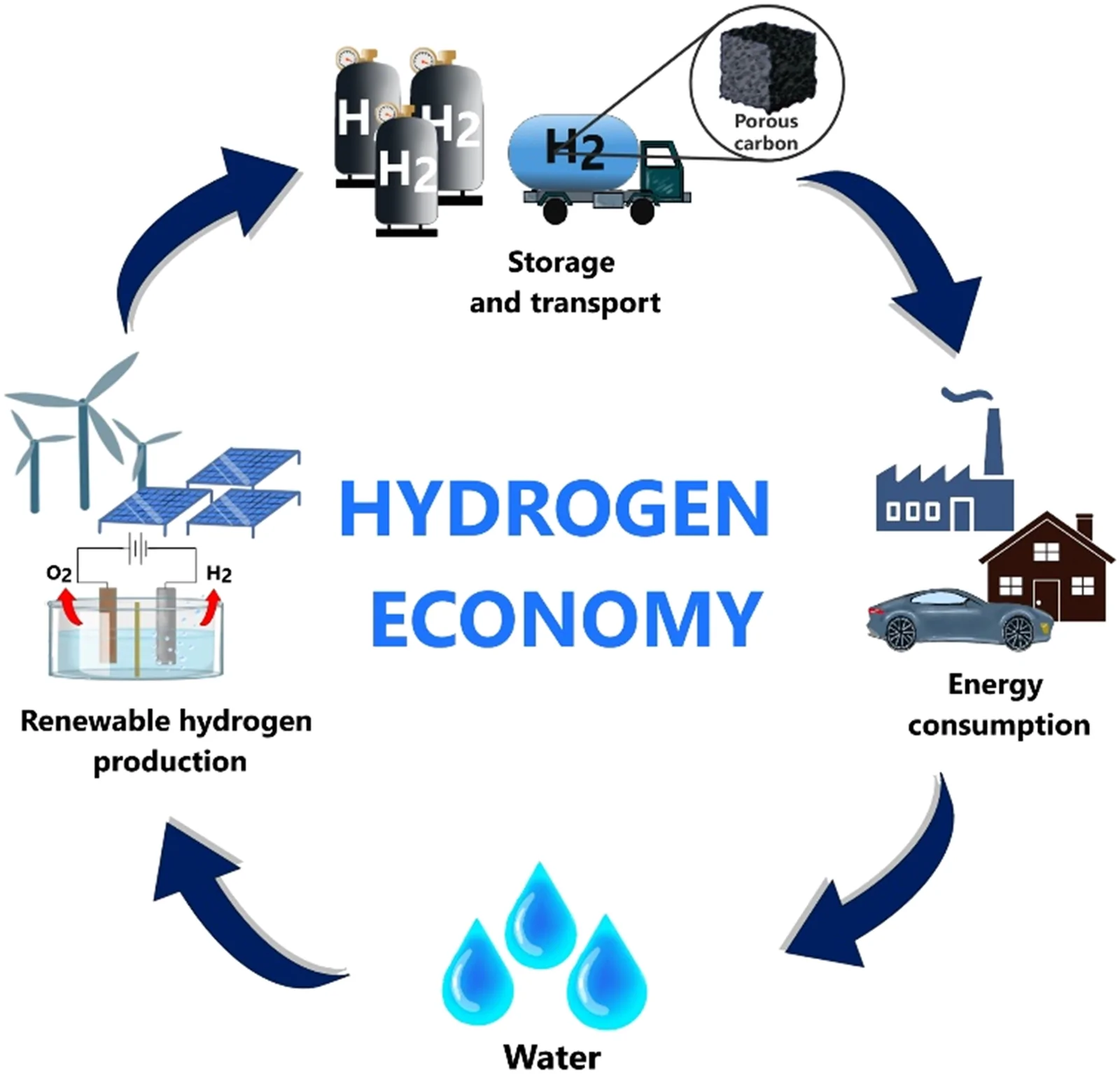
Schematic showing the hydrogen cycle as part of hydrogen economy.

The development of sustainable and efficient energy systems is crucial for meeting the global demand for clean energy. Our increasing consumption of fossil fuels contributes to the release of greenhouse gases into the atmosphere, leading to global warming and climate change.^1^ In addition, limited reserves, uneven distribution, and price instability of fossil fuels are increasingly becoming driving forces for many countries and economies to turn to renewable sources.^2^ Hydrogen is a promising energy vector for replacing fossil fuels as it is potentially renewable and clean,^3^ producing only water, heat and energy when used in a fuel cell. Additionally, hydrogen has the highest gravimetric energy density (energy stored per unit mass) of any commonly used fuel, at 142 MJ kg^−1^, compared to 44 MJ kg^−1^, 53.6 MJ kg^−1^, and 45.4 MJ kg^−1^ for gasoline, natural gas, and diesel, respectively,^4,5^ making it one of the most efficient and sustainable sources of energy.^6^ Despite the positive aspects of using hydrogen, a successful transition to a hydrogen-powered economy requires the remediation of some of its downsides for practical use (as shown in Scheme 1). For example, hydrogen has a very low critical temperature of 33 K and under ambient conditions it exists as a gas with a very low density of 0.083 kg m^−3^.^7^ This combination of low gas density and extremely low critical temperature makes it highly challenging for developing compact high-density hydrogen storage systems. To overcome this problem, it is crucial to find solutions to increase the volumetric energy density (energy storage per unit volume) of hydrogen for practical applications. As a guideline, based on the requirements for light-duty vehicles with an ultimate driving range of more than 500 miles, the US Department of Energy (US DOE) has established goals for gravimetric and volumetric hydrogen storage capacities for on-board vehicle applications while maintaining safety, cost and performance requirements (Table 1).^8^

**Table 1:** US DOE targets for gravimetric and volumetric hydrogen storage capacity for on-board vehicle applications. Taken from ref. 9

Storage parameter	Units	2020	2025	Ultimate
System gravimetric capacity				
Usable, specific energy from H_2_ (net useful energy/max system mass)	kW h kg^−1^ (kg H_2_/kg system)	1.5 (0.045)	1.8 (0.055)	2.2 (0.065)
System volumetric capacity				
Usable energy density from H_2_ (net useful energy/max system volume)	kW h L^−1^ (kg H_2_/L system)	1.0 (0.030)	1.3 (0.040)	1.7 (0.050)

The US DOE identified that maximising hydrogen storage capacity is the one of the essential factors to be taken into consideration for successful utilization of hydrogen for on-board applications. However, other factors like operating cost, durability/operability, and charging and discharging rate, and safety have also been identified with defined targets. In a report published by U.S. DRIVE (Driving Research and Innovation for Vehicle efficiency and Energy sustainability) the ultimate cost for an on-board hydrogen storage system is set at $8 USD per kW h.^8^ Other goals include operability under ambient conditions in a range of −20 °C and 60 °C and minimum and maximum system delivery pressures of 5 to 20 bar (absolute), an ultimate operational life cycle of 1500 times, quick refill, and a target of more than 90% energy efficiency for hydrogen storage systems.^8^ Clearly, the development of hydrogen storage materials that meet these goals is a top priority to realize the targets set by the US DOE. In order to achieve these targets, researchers and engineers are exploring a wide range of potential storage materials and methods. Currently, several types of hydrogen storage technologies are being studied,^10^ such as (i) compression in high-pressure cylinders, (ii) liquification of hydrogen at cryogenic temperatures, (iii) adsorption into porous materials with high surface areas, (iv) interstitial adsorption of hydrogen on metals and clathrates, (v) *via* liquid organic hydrogen carriers, (vi) using complex hydrides, (vii) precursors with binding of hydrogen through the formation of covalent or ionic bonds, (viii) chemical precursors like ammonia that can decompose to hydrogen and (ix) *via* the oxidation of reactive metals in the presence of water. Physical storage methods include compression and liquefaction. Compression is the most popular method of hydrogen storage for on-board applications.^11^ However, despite being a highly developed method of storage, compression can be economically unfavourable as storing hydrogen under high pressures (often up to 70 MPa) can compromise energy equivalent to 13–18% of its lower heating value.^6,12^ Additionally, safety concerns and practical limitations become critical as a result of limited durability of pressurised tank materials. In addition, any further increase in volumetric capacity for hydrogen would require further increases in pressure, but that would result in compromising gravimetric density, due to the need for thicker tank walls that increase the weight and cost of the storage system.^10,13^ For liquefaction, hydrogen is usually stored at temperatures below 30 K at ambient pressures and requires well-insulated storage tanks. Both methods have proven to be energy-intensive and costly. Standards established for hydrogen storage tank systems for light-duty, heavy duty and ground storage applications require use of expensive materials like composites of carbon fibres, polymer liners, and steel.^14,15^ Therefore, material-based approaches for efficient hydrogen storage have attracted much attention during the past two decades, as they offer solutions to the limitations set by the two aforementioned methods.

**Fig. 1:**
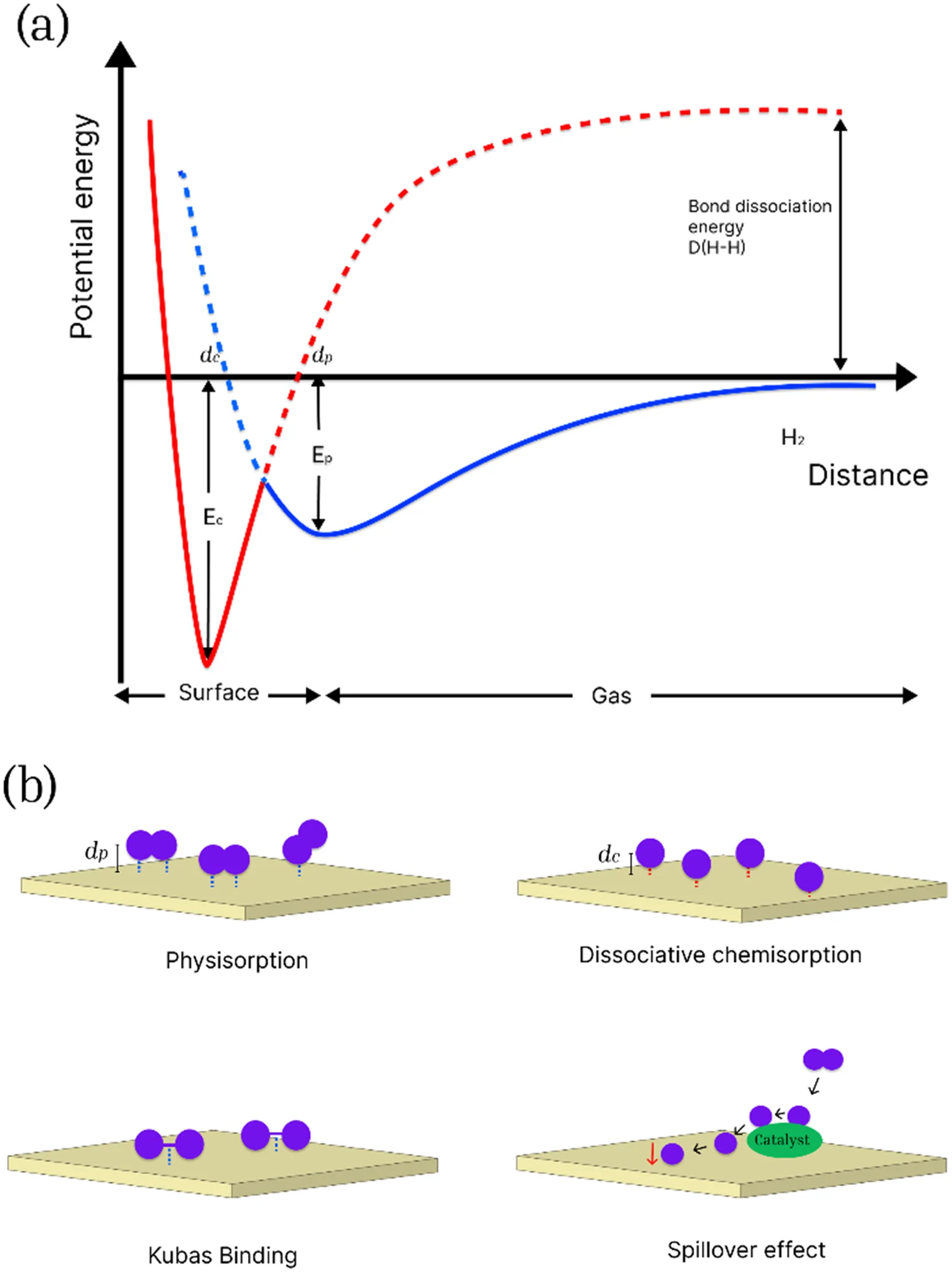
(a) Potential energy curves for physisorbed and chemisorbed hydrogen as a function of distance *d* from the surface of the adsorbate. Adapted from ref. 17; (b) a schematic showing the different types of mechanisms for hydrogen adsorption on an adsorbate surface.

Storage into solid-state materials depends on the material–hydrogen interactions, and mainly involves physical, chemical and intermediary interactions. Physisorption or physical adsorption is an exothermic non-specific process in which molecular hydrogen is adsorbed on the surface of the material *via* weak van der Waals forces.^16^ The overall combination of short-range repulsive forces between H_2_ molecules and long-range attractive forces results in a relatively shallow and small minimum in the potential energy curve (shown in the Lennard-Jones potential diagram in Fig. 1a). Due to this spontaneous interaction, there is no activation energy barrier to facilitate the adsorption of hydrogen on the surface of the adsorbent, giving rise to the fast kinetics of physisorption.^17,18^ Physisorption involves low enthalpies of adsorption (<10 kJ mol^−1^) and it is inversely related to ambient temperature, hence it is necessary to maintain cryogenic temperatures for efficient storage.^16,19^ Physisorption is most effective in porous materials with high specific surface areas (SSA), including MOFs, porous carbon, covalent organic frameworks (COFs), and organic polymers. Chemisorption, on the other hand, is an activated process that involves the selective adsorption of the adsorbate on the active sites of the adsorbent through the formation of strong chemical bonds.^20^ The enthalpy of chemisorption on metal surfaces is between 40 and 160 kJ mol^−1^.^21^ The potential energy curves for chemisorbed and physiosorbed hydrogen are shown in Fig. 1.

Two minima are shown at distances *d*_c_ and *d*_p_, corresponding to the equilibrium distance for chemisorption and physisorption, respectively. In chemisorption hydrogen is absorbed into the crystal lattice of the adsorbent, such as metals to create a hydrogenated metallic phase at ambient temperatures and pressures, resulting in dissociation into its atomic form and the formation of metal hydrides or complex hydrides.^22^ However, despite high storage uptake chemisorption predominantly suffers from slow kinetics and desorption due to the presence of an activation barrier. High temperatures are needed to release the stored hydrogen due to the high binding energy of hydrogen to the substrate. For example, a high adsorption capacity of 8.7 wt% has been achieved for lithium–beryllium hydrides with sorption and desorption temperatures of 300 °C and 320 °C, respectively.^23,24^ Various types of materials that store hydrogen by chemisorption have been studied for hydrogen storage, including widely studied metal hydrides like MgH_2_ and LiBH_4_.^25,26^

**Fig. 2:**
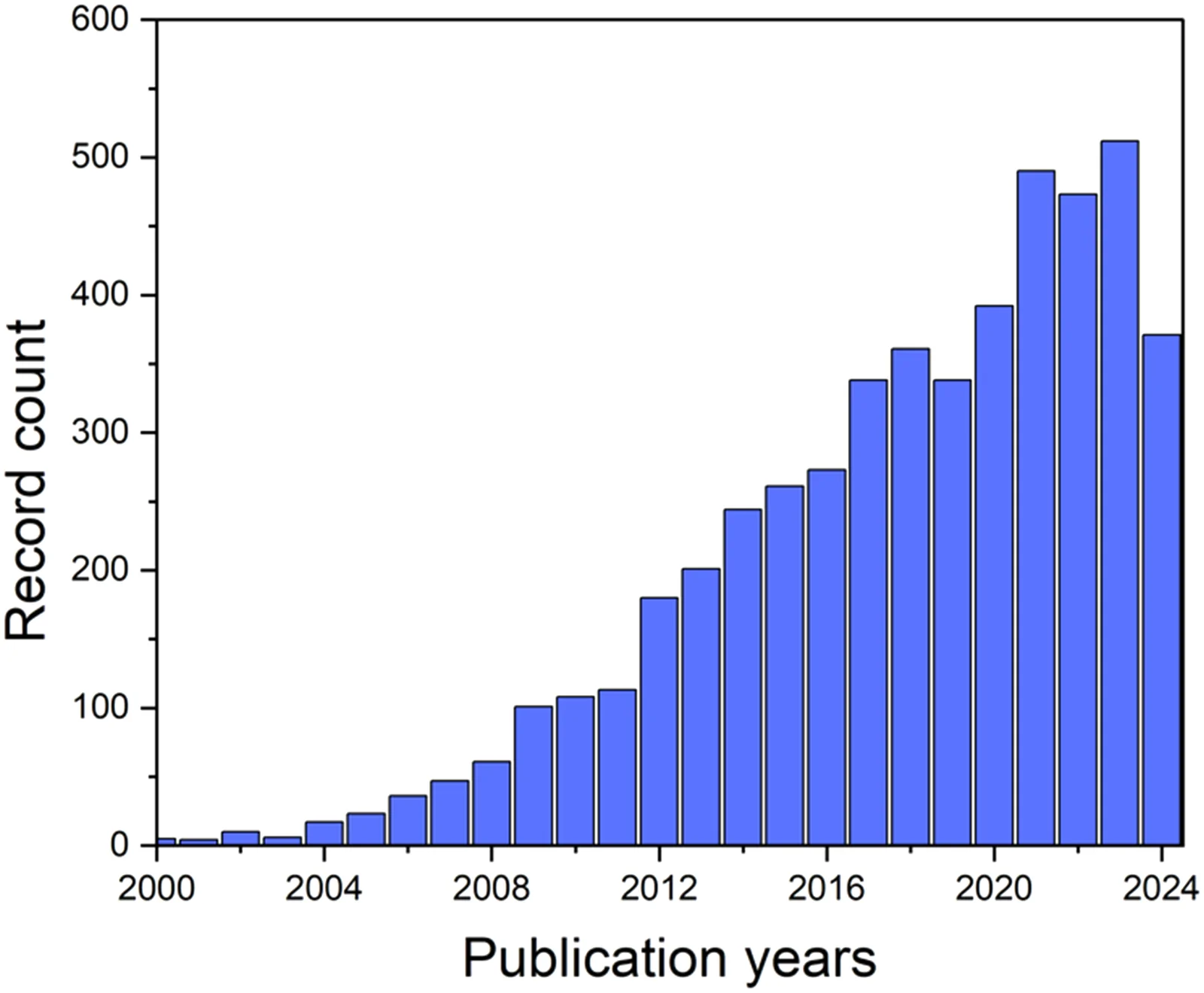
Graph showing the rising trend in the number of publications per year based on a search for “porous carbon” and “hydrogen storage” on Web of Science. Accessed on 26th of September 2024.

Further modifications of solid-state materials have prompted study into other proposed intermediary adsorption and storage methods *via* hydrogen spill-over and quasi-molecular interactions (otherwise known as Kubas binding) which are shown in Fig. 1.^27^ In these proposed processes, the presence of a metal catalyst or a heteroatom on the surface of porous materials is believed to facilitate adsorption by increasing binding enthalpies and therefore, can contribute towards improved adsorption kinetics and hydrogen storage capacity of the materials.^28^ Kubas binding involves the donation of σ-bonding electrons to an empty d-orbital and π back-donation from a filled d-orbital into the empty σ* anti-bonding orbital of hydrogen, hence causing elongation of an H–H bond.^29^ For practical applications, the primary advantages of hydrogen storage by physisorption are the rapid adsorption and desorption kinetics and more practical operating conditions when compared to chemisorption.^30^ Among porous materials that are widely studied, MOFs an COFs can be advantageous in their tuneable structure, highly crystalline nature, and they can be synthesised fairly consistently. The very high surface area makes these materials attractive for hydrogen storage applications. In addition, the presence of metal binding sites and the ability to post-synthetically modify the linkers in MOFs provide additional advantages over other types of porous materials. However, the major caveat to using MOFs, COFs and porous polymers like PIMs is that despite the high surface area, they can be limited by relatively poor chemical and thermal stability, and challenges involved in sustainable large-scale synthesis. Porous carbon on the other hand can be a cost-effective alternative. They can be more easily synthesised in large industrial scales and can be sourced from recyclable carbon-rich waste materials, including bio-based precursors. Porous carbons have attracted much attention recently (Fig. 2), as they offer several advantages over other materials. They also offer a multitude of optimization strategies for improving hydrogen storage capacity due to their high chemical and thermal stability compared to other materials, such as MOFs and porous polymers.^31–35^ The following table (Table 2) provides a comparative overview of various types of porous materials for hydrogen storage applications, highlighting the benefits and drawbacks of these materials based on different parameters.

**Table 2:** Comparison different important parameters of commonly used porous materials for hydrogen storage applications

Parameter	MOFs	COFs	PIMs	Porous carbon
Porosity	Very high, tuneable pore sizes and shape (*e.g.* ranging from 2 to more than 50 nm)^36^	High porosity. Tuneable pore sizes	Moderate porosity. Tenable porosity	High porosity, though not as tuneable as MOFs. Non-uniform pore sizes
Surface area	Extremely high surface area (*e.g.* experimental highest BET SSA for NU-110, 7140 m^2^ g^−1^. Theoretical upper limit of 14 600 m^2^ g^−1^)^37^	High surface area (surface area can reach 5000 m^2^g^−1^)^38^	Moderate surface area (around 1000 m^2^ g^−1^)^39^	High surface area (*e.g.* highest experimental surface area for activated carbon is 3839 m^2^ g^−1^)^40^
Thermal and chemical stability	Moderate chemical and thermal stability^41^	Moderate thermal and chemical stability^42^	Moderate thermal stability (*e.g.* PIM-7 had thermal decomposition temperature of 480 °C;^43^ PIM-1 retained stability for 400 days with loss of 0.7 wt% at 77 K and 100 bar)^44^	Very high chemical and oxidative stability^45^
Synthesis	Variety of synthesis methods. Involving building blocks, metal salts and organic linkers (*e.g.* solvothermal, microwave-assisted, mechanical)^46^	Variety of synthesis methods. Complex synthesis and processability. Difficulty to synthesize on a larger scale (microwave, solvothermal, mechanical)^38^	Moderate synthesis difficulty^47^	Variety of synthesis methods. Accessible and cheaper sources of precursor (*e.g.* biomass). Activation methods can involve chemical or physical methods^48,49^
Hydrogen storage capacity	High hydrogen storage capacity (highest gravimetric total capacity reported for DUT-32 is 14.21 wt% at 80 bar and 77 K;^50^ highest gravimetric excess capacity reported for NU-100 is 9.13 wt% at 56 bar and 77 K)^51^	High storage capacity (COF-103 has gravimetric hydrogen storage capacity of 6.6 wt% at 35 bar and 77 K)^52^	Moderate H_2_ uptake of 1.7 wt% (77 K, 10 bar)^39^	High hydrogen storage capacity (*e.g.* excess gravimetric storage capacity is 7.0 wt% at 77 K and 20 bar)^53^
Functionalisation	Easy to functionalise by post-synthetic, or *in situ* modifications.^54^ Presence of metal binding sites for adsorption	Lack of H_2_ binding sites. Potential for modification^55,56^	Lack of binding sites, potential for functionalisation by post synthetic modification^57–59^	Lack of H_2_ binding sites. Can be functionalised by heteroatom doping and metal nanoparticles
Environmental impact	Presence of toxic starting materials, solvents and waste products. Can be synthesised by mechanochemical methods	Use of solvents and energy in conventional solvothermal methods	Wet chemical methods use solvents like DMSO and toluene. Process can be energy intensive.^60^ Can be synthesised by mechanochemical methods	Moderate environmental impact. Depends on the synthesis method and choice of precursor. Biomass derived carbon can be sustainable and green
Cost	High cost due to use of expensive organic linkers, metal salts, solvents, as well as processes such as heating and drying^61^ (*e.g.* synthesis costs could range from 35–71 and 13 to 36 USD per kg for solvent-assisted and solvent-free synthesis, respectively)^61^	Solvothermal synthesis can be energy intensive and uses a large amount of solvents, and expensive COF monomers^62^ therefore costly. However, some environment friendly synthesis methods (*e.g.*, mechanochemical) are available	High cost due to use of large amounts of organic solvents	Can be cost effective by choosing the precursors and activation method

In this review, we will briefly outline different types of porous carbon materials for hydrogen storage, with an emphasis on their synthesis methods and strategies for improving storage capacity and operating conditions. Lastly, we will outline the future prospects of incorporating carbon-based materials into a sustainable hydrogen economy, by presenting the challenges and potential for their use as future hydrogen storage materials.

## 2. Properties of porous materials influencing H_2_ storage by physisorption

**Fig. 3:**
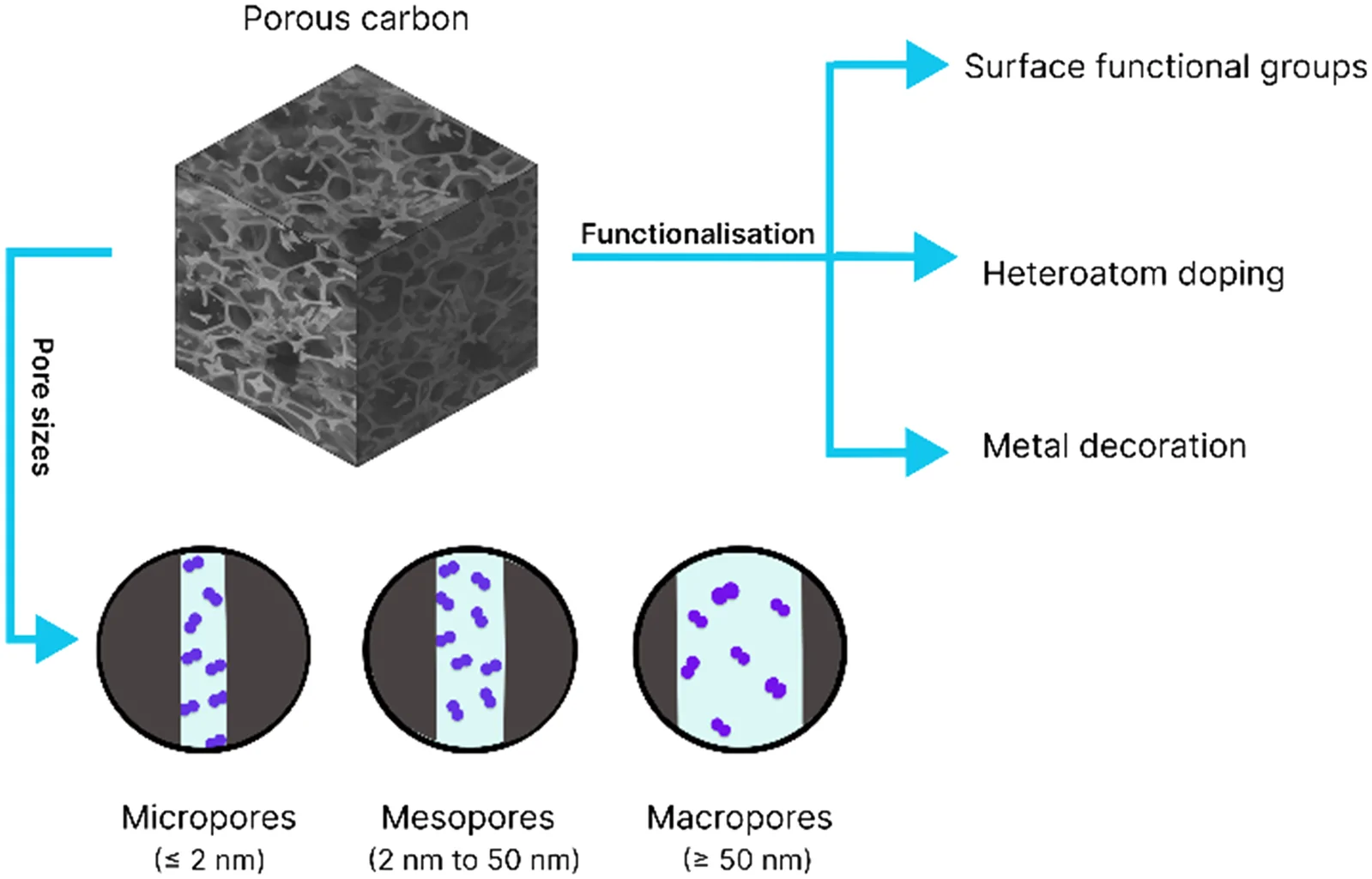
Schematic showing the structure property relationship of porous carbon, the pore size and types of functionalization, which are important factors in enhancing hydrogen uptake in carbon-based sorbents.

The physical characteristics of porous carbon play a critical role in determining hydrogen uptake capacity (shown in Fig. 3). Firstly, as physisorption is a key factor in H–adsorbate interactions on porous carbon, the SSA is an important parameter for improving the hydrogen uptake capacity of porous materials. Typically, a higher SSA means more adsorption sites are available for hydrogen to interact with the surface. As a rule of thumb, materials exhibit a 1 wt% increase in gravimetric H_2_ uptake per 500 m^2^ g^−1^ SSA (measured at 77 K). This correlation is well-known in the community as Chahine's rule.^9^ However, porous materials frequently surpass this due to another important factor, the pore size, as we will discuss next. Secondly, although there is a relationship between the surface area and hydrogen uptake, it is important to also consider the interrelation between the surface area and pore volume in controlling hydrogen storage capacity. The size and shape of the pores greatly affect hydrogen interactions, by influencing hydrogen's accessibility to adsorption sites of the material and the strength of the interactions. Based on pore diameter IUPAC has defined three types of pores: macropores (pore diameter ≥ 50 nm), mesopores (pore diameters between 2 nm and 50 nm), and micropores (pore diameter ≤ 2 nm).^63^ Typically, micropores provide the optimal size for better hydrogen adherence to the adsorbent walls. The pore sizes are small enough to result in the overlap of potential fields of opposing pore walls, resulting in the increase of its isosteric enthalpy of adsorption, hence adsorbing hydrogen at much higher densities than its gaseous form,^64,65^ with a reported density of up to 100 kg m^−3^.^66^ It has been found that the pore diameter for maximal hydrogen uptake ranges from 0.6–0.7 nm at elevated pressure and 77 K.^67^ A smaller pore size than this would not allow dynamic hydrogen atoms to be contained within the pores, and a larger one will decrease the interaction between the molecular hydrogen and pore walls. A number of studies have led to this conclusion, by demonstrating optimal hydrogen uptake within the narrow range of pore size distribution (PSD) below 1 nm.^68^ Hence, it is essential to consider the importance of the PSD when developing porous materials for hydrogen storage applications. Another important parameter for hydrogen storage capacity is the micropore volume. It is a measure of the extent of the material's microporosity which is determined from the cumulative volume of micropores. There is a positive direct relationship between micropore volume and hydrogen storage capacity, with higher micropore volumes in porous materials correlating to increased hydrogen uptake capacity.^69^

Another important parameter that directly influences the H_2_ storage capacity is the isosteric enthalpy of adsorption. It is defined as the amount of heat released when an adsorbate binds to an adsorbent. It is also referred to as the isosteric heat of adsorption.^70,71^ However, many researchers have recommended the use of enthalpy of adsorption over the heat of adsorption, as enthalpy represents the thermodynamic change. A higher magnitude of enthalpy corresponds to stronger adsorbent–adsorbate interactions, and hence, more hydrogen will be adsorbed at a given temperature and time.^72^ In porous carbon materials, the enthalpy of adsorption of hydrogen is relatively low, ranging from −4 to −6 kJ mol^−1^, as it relies on physisorption of hydrogen to the surface *via* weak van der Waals interactions.^73^ The optimal enthalpy of adsorption for hydrogen storage is between −15 and −20 kJ mol^−1^ under ambient conditions.^74^ Such isosteric heat of enthalpies can be achieved by optimizing the conditions for adsorption, chemical, and physical characteristics. Surface functionalization plays an important part in enhancing hydrogen uptake in porous carbon materials. Modifying the surface chemistry with heteroatoms, metal nanoparticles, and other functional groups has been proven to increase the interactions of hydrogen with the materials. For example, porous carbons doped with Ni, Fe, or Mn have shown an increased magnitude of enthalpy of adsorption to 6.52, 6.31 and 6.14 kJ mol^−1^, respectively, as opposed to 6.07 kJ mol^−1^ for undoped porous carbon. This indicates increased H_2_–adsorbate interactions.^75^ These functional groups and metal nanoparticles facilitate stronger adsorption by providing additional binding sites for hydrogen atoms and by creating localized regions of polarity through dipole–dipole interactions.^76^ Surface functionalization with metal catalysts like Pt and Pd modifies the electronic structure of the carbon material, resulting in charge redistribution and transfer, hence giving rise to a catalytic phenomenon called the spillover effect.^9,77^ First reported in 1964, the spillover effect is defined as the migration of hydrogen atoms catalysed by a metal nanoparticle to an H^+^ accepting site on the catalyst support while simultaneously reducing the metal sites.^77,78^ Temperature programmed reduction (TPR) experiments conducted by Briggs *et al.* have postulated the mechanism and its effect in enhancing hydrogen storage capacities using Pd and CuO catalysts on a carbon nanotube support.^79^ Further experiments as well as DFT studies have supported the spillover effect as a potential mechanism for enhanced hydrogen storage.^80^ Although the exact mechanism for the spillover effect is still debated, it has been proposed as a primary mechanism for enhanced hydrogen uptake capacity of porous materials at room temperature in multiple studies.^81–84^

### 2.1. Practical hydrogen storage

In order to successfully incorporate porous materials as a suitable and practical method for hydrogen storage, a number of parameters need to be considered. The term practical hydrogen capacity assesses the potential of materials for widespread applications. This includes factors like stability, cost, ease of synthesis/fabrication, and safety. Additionally, it also includes factors relating to its performance, like reversibility (kinetics), recyclability, adsorption capacity, and thermodynamic properties, which govern the operating conditions for efficient storage and delivery.

#### 2.1.1. Adsorption measurement

**Fig. 4:**
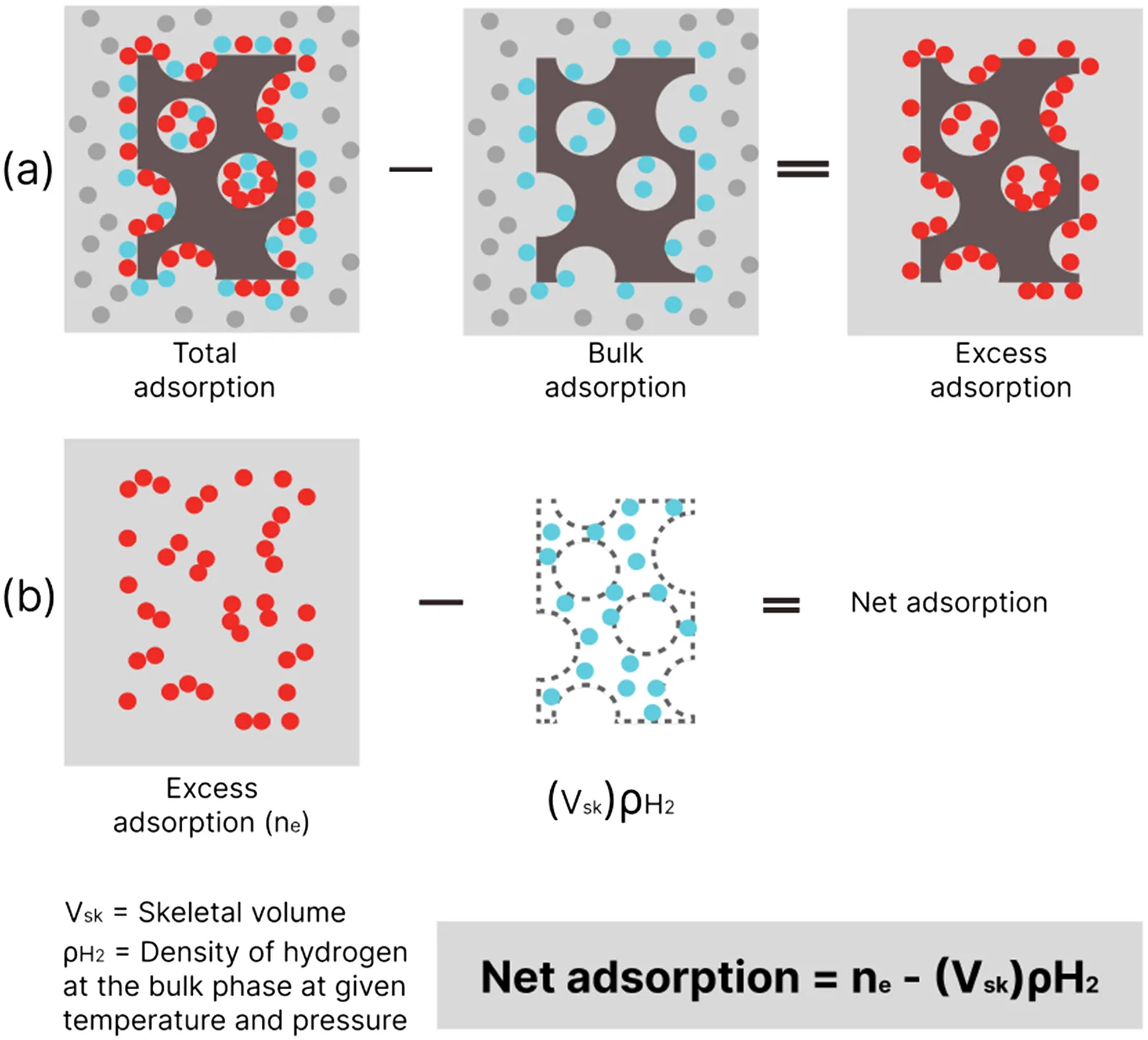
Scheme illustrating (a) excess adsorption and (b) net adsorption. Grey dots represent the free hydrogen molecules and coloured dots represent the adsorbed hydrogen molecules. Blue dots in (a) are hydrogen molecules of the bulk phase that would be present without interaction between the molecules and adsorbent. Red dots represent the hydrogen molecules adsorbed on the surface due to interaction. Blue dots in (b) represent the amount of hydrogen in the bulk phase that would occupy the skeletal volume under the given experimental conditions.

In the literature, most studies for hydrogen storage are reported in terms of gravimetric and volumetric capacity. Gravimetric capacity (*C*_wt%_) is defined as the amount of hydrogen stored per unit mass of material, and is usually reported in H_2_ kg kg^−1^ or as a percentage ratio of weight. As shown in eqn (1), the gravimetric capacity, *C*_wt%_, of a material can be expressed as a ratio of the mass of adsorbed hydrogen over the total mass of the adsorbent–adsorbate system, where *n*_e_ and *n*_Host_ are the number of moles of excess (known as the Gibbs surface excess) adsorbed hydrogen and the sample material, respectively.1
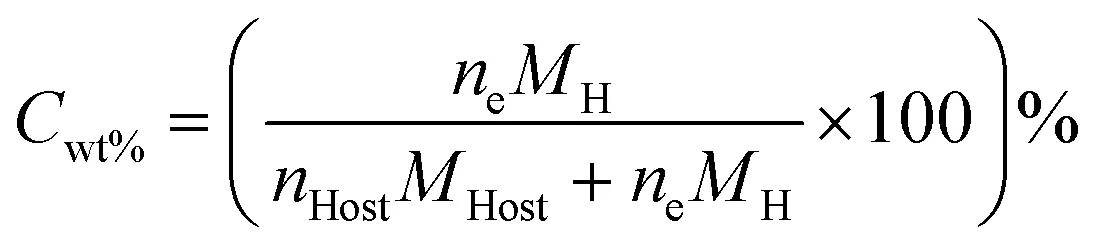
while *M*_H_ and *M*_Host_ are the molar masses for hydrogen and the adsorbent material, respectively. The excess adsorbed hydrogen is defined as the amount of hydrogen stored at higher densities on the Gibbs surface of the adsorbent compared to the free-gas phase in the bulk (Fig. 4).^85^ However, as gravimetric measurements rely on weight to measure adsorbed hydrogen, they are only sensitive to the excess and do not reflect the total amount of hydrogen that is present inside the pores. Hence, another representation of capacity known as absolute and total capacities has been studied. This leads to a series of assumptions to be taken into consideration for the conversion of excess to total (*n*_tot_) or absolute (*n*_abs_) capacity as shown in eqn (2) and (3), respectively.^86^ Absolute capacity is defined as the total amount of hydrogen stored within the adsorbate phase, where *P* is the pressure at equilibrium, (*M*) is the molar mass, (*R*) is the gas constant and (*T*) is the temperature.^87^ Most studies assume that the gaseous phase of adsorbed hydrogen (*ρ*_ads_) is equivalent to liquid hydrogen, at approximately 70 kg m^3^.^86^ However, it has been estimated that porous carbon materials exhibiting high micropore volumes can store hydrogen at much higher densities, estimated to reach ∼100 kg m^3^ at 77 K.^66^2
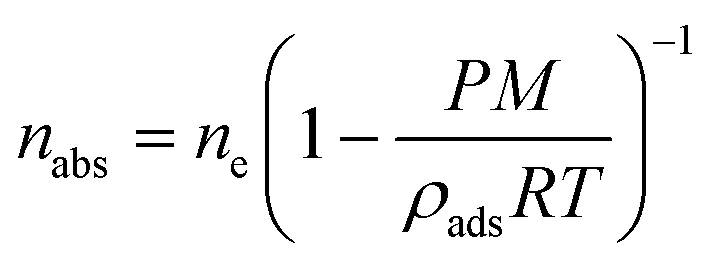
3
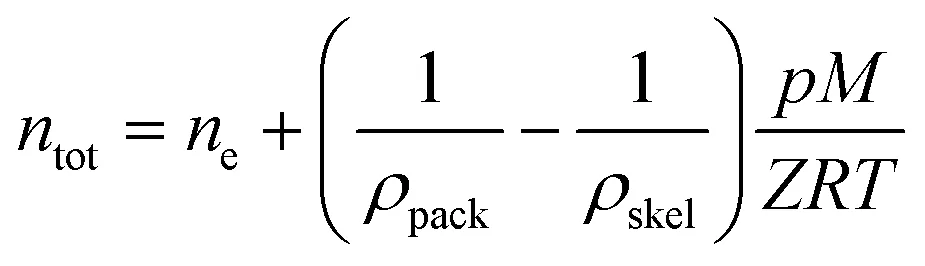
Total hydrogen capacity (*n*_tot_) is the total amount of hydrogen in the adsorbate. This requires the introduction of other definitions of density including packing density (*ρ*_pack_) and skeletal density (*ρ*_skel_). These density parameters allow for a more accurate evaluation of material performance.^88^ However, there are some ambiguities surrounding the definition of total adsorption. As it is difficult to calculate the free gas volume inside the adsorbate phase, a more definitive parameter, net adsorption (*n*_net_), was introduced which is defined as the difference between the total amount of gas in a system in the presence of an adsorbent and without it.^89^ Net adsorption helps to give a clearer comparison to assess the benefit of using an adsorbent in a hydrogen storage vessel over an empty vessel without any adsorbent (Fig. 4).^88^ For this reason, net capacity is also referred to as “engineering capacity”. If the stored amount of hydrogen in the space occupied by the adsorbent itself (skeletal volume, *V*_sk_) would be more than the stored amount of hydrogen in the presence of the adsorbent under a given condition, the net capacity for the adsorbent becomes negative. This makes the storage system inefficient compared to an empty vessel. Therefore, net capacity is information that can be used to evaluate if there is any extra advantage of using an adsorbent for storage applications. Net adsorption also contains less uncertainty as it doesn't rely on measurements to determine the volume of gas adsorbed, as was highlighted by Broom *et al.* recently.^90^ Despite its benefit for practical applications, net adsorption is not widely reported in the literature.

Volumetric capacity is defined as the amount adsorbed per unit volume, measured in units of H_2_ kg m^−3^. Although it gives useful insight on the amount of hydrogen adsorbed, it assumes that the volume doesn't change with adsorption of hydrogen. As with gravimetric storage, it is difficult to determine the exact amount of adsorbed hydrogen, and hence, an assumption is made that the adsorbed hydrogen occupies all the pores in the material. This is called the *total pore approximation*.^91^

Both methods of quantifying the adsorbed hydrogen can provide useful insight into the material's characteristics and its ability to store hydrogen. At low temperatures, the trend in improving the gravimetric storage capacity of porous materials seems to be correlated with the BET surface area, as highlighted previously in Chahine's rule. Based on this correlation, research has been focused on microporous materials to develop materials with ultrahigh surface areas to achieve high hydrogen storage capacity. MOFs like NU-100 with a reported BET surface area of around 6143 m^2^ g^−1^ showed an excess gravimetric capacity of 9.95 wt% at a temperature of 77 K and pressure of 5.6 MPa.^51^ However, efforts to improve gravimetric capacities alone might not be the optimal strategy for practical hydrogen storage.^92^ Other metrices, such as the volumetric hydrogen storage capacity, also need to be taken into consideration. Volumetric measurements require knowledge of the material's bulk density or packing volume, and hence, are correlated with the surface area occupied per unit volume, known as the *volumetric surface area*. This factor can be optimised by either reducing the bulk volume, or enhancing the surface area, using various approaches including powder compression, incorporation of porous materials into monoliths or polymer composites,^44,93^ and enhancing interpenetration in the case of open framework materials like MOFs.^94^

#### 2.1.2. Usable capacity and thermodynamics

Another important factor to take into consideration is usable capacity, which is the amount of hydrogen that can be delivered within the limits of operating ranges of temperature and pressure in a system/material, as defined by thermodynamic restrictions.^95,96^ This means that the amount of usable/deliverable hydrogen at tank temperature (*T*) depends on the difference in hydrogen capacities between the maximum tank pressure at equilibrium (storage pressure *P*_i_) and the minimum back pressure (exhaust pressure, (*P*_bk_)), as defined by eqn (4).^96^

4 *n*_uc_(*P*_i_) = *n*_abs_(*P*_i_) − *n*_abs_(*P*_bk_)

In addition to pressure, the usable capacity is also governed by temperature. The temperature at which the maximum amount of hydrogen is released is called the optimum operating temperature. As shown in eqn (5), the optimum operating temperature (*T*_opt_) is governed by the isosteric enthalpy of adsorption (Δ*H*°) which is an indicator of the strength of adsorbate–adsorbent interactions,^74^ Δ*S*° is the entropy change relative to standard pressure (*P*_0_), *P*_1_ is the storage pressure, and *P*_2_ is the exhaust pressure. A higher enthalpy of adsorption results in a higher operating temperature.5
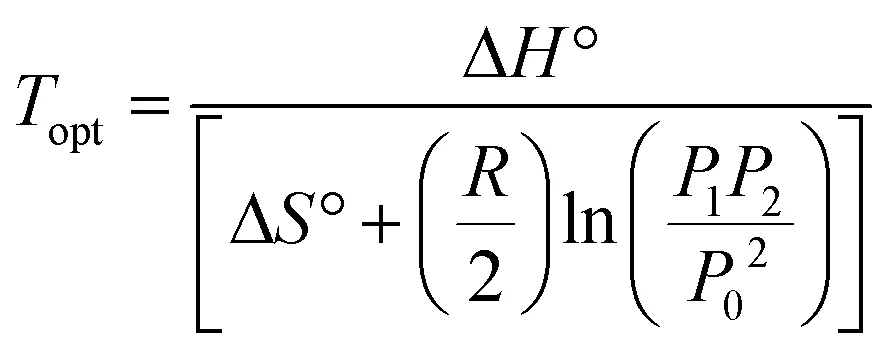
Governed by the above correlation, material development efforts have been directed towards improvement of the operating temperature of materials by increasing the isosteric enthalpy of adsorption. For carbon materials, the enthalpy of adsorption is usually −5.8 kJ mol^−1^. Through thermodynamic calculations, it can be determined that the optimal operating temperature for carbon is about 115 K between 30 bar and 1.5 bar pressure.^74^ As discussed earlier, an isosteric enthalpy of adsorption of −15 to −20 kJ mol^−1^ is particularly desirable for efficient delivery at ambient temperatures and pressures from 30 to 1.5 bar. Although carbon materials show relatively low isosteric enthalpy of adsorption due to the low strength of the adsorbate–adsorbent interactions between hydrogen molecules and carbon, they can be modified to improve interactions to reach the desired range for practical applications. The thermodynamic restrictions are hence what governs the operating conditions for sufficient hydrogen adsorption–desorption cycles. Higher enthalpy of adsorption results in higher operating temperature for degassing.^96^ Hence there's a trade-off between optimising operating conditions and storage capacity.

**Fig. 5:**
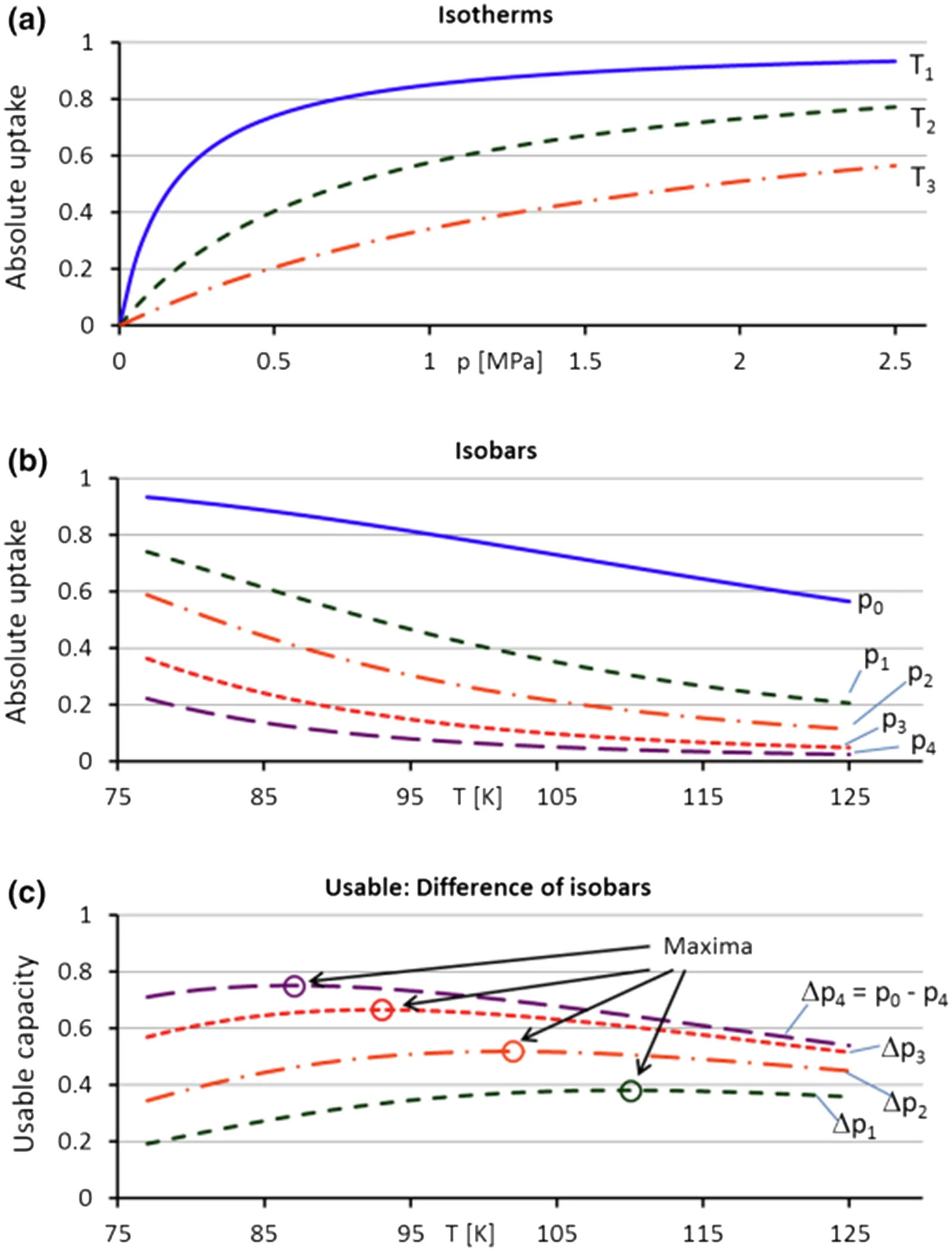
Plots of absolute uptake and usable capacity of hydrogen against various temperatures and pressures showing: (a) typical type I adsorption isotherms plotted at various temperatures, *T*_1_, *T*_2_ and *T*_3_. (b) Usable capacity when plotted at isobars for various pressures, *P*_0_, *P*_1_, *P*_2_, *P*_3_, and *P*_4_ at different temperatures. (c) Isobars with the usable capacity as the change in absolute adsorption at difference in pressure Δ*P*. Reproduced with permission from ref. 96.

Based on the IUPAC classification, microporous carbon materials show type I (a) and type I (b) adsorption behaviour at 77 K,^97^ meaning that the equilibrium uptake of hydrogen initially increases with increasing pressure, until a plateau or a saturation point is reached at higher pressures. The adsorption isotherm of a material relates to its potential for reversible hydrogen storage. Usable storage capacity is the amount of hydrogen that can be adsorbed and released within lower and upper limits of operating pressure. As shown in Fig. 5, the usable capacity can be visualised as isobars plotted by calculating the difference between the upper and lower pressure limits of pressure at a given temperature. In Fig. 3(c), the usable capacity is maximum at the optimum operating temperature, and as it is shown earlier in eqn (5), its value is dependent on the upper and lower pressure limits.

Another important quality of the material is its cycling stability. It is the ability of the adsorbate material to recover its hydrogen storage capacity along hydrogen charging and discharging cycles.^98^ Recyclability depends on the chemical and thermal stability of the material at the temperatures of hydrogen cycling. However, it can be negatively impacted by impurities present in the gas as well; adsorption of contaminants is more likely to reduce the hydrogen storage capacity, due to favoured adsorption of gaseous contaminants including water and nitrogen onto the adsorption sites.^99,100^

## 3. Porous carbon

**Fig. 6:**
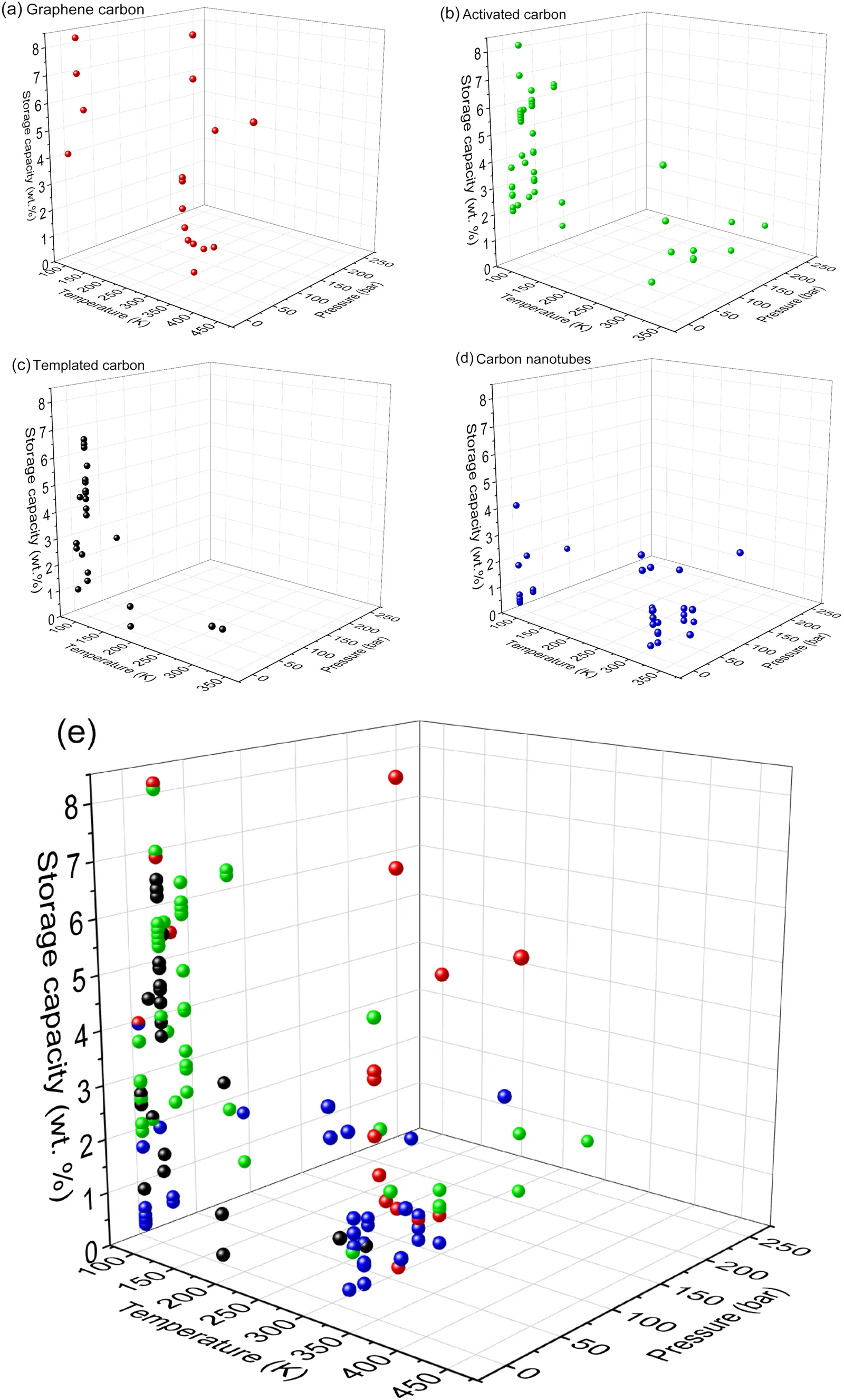
Scatter plot of temperature (K) and pressure (bar) *versus* excess storage capacity (wt%) for (a) graphene materials, (b) activated carbon, (c) template-derived carbons, and (d) carbon nanotubes taken from reported data. (e) Overlay of scatter plots (a)–(d). Plotted from the data reported in references listed in Table 3.

As mentioned earlier, porous carbons are a promising class of materials for hydrogen storage due to their high surface area, functionalization, high stability, and tailorable pore size. Additionally, porous carbons are a group of materials with various structural diversity, starting from the simplest 2-D sheet-like structure of graphene to the complex and interconnected network of activated carbons. These groups vary greatly in their structural morphology, including the surface area and PSD, in addition to their synthesis methods and precursors. For example, it has been shown that activated carbon can be sourced from biomass waste materials which makes it an economically favorable material to synthesize. Template-derived carbons made from carbide and zeolite precursors, and activated carbons have shown the highest hydrogen storage capacity to date, showing a maximum hydrogen storage capacity of 6.9 wt% and 8.1 wt%, respectively, at 2 MPa and 77 K.^101,102^ This is primarily due to their high micropore volume and SSA, as a result of their tuneable synthesis and activation methods. Moreover, other types of carbon have been explored, including graphene and graphene-based materials like graphene and nanotubes, with an estimated theoretical hydrogen storage capacity of 7 wt% at 77 K and 1 MPa, and with computer simulations, respectively. The high volume of research conducted on different classes of porous carbon for hydrogen storage is reflected in the number of papers published (shown in Table 3). Additionally, Fig. 6 presents a 3D-scatter plot of different types of carbon materials. As can be observed, most studies are carried out at 77 K and 20 bar and ambient temperatures. From this comparative plot it can be seen that graphene, activated carbons, and template derived carbons occupy high ranges of uptake capacity under these operating conditions. The comparison also indicates a general trend of much lower hydrogen uptake of carbon nanotubes than the other carbon materials. Additionally, dispersion of the uptake capacity can reflect on the reproducibility of the results, as activated carbons can reach high surface areas and a bigger range of pore size distribution. This can make observations like Chahine's rule more prominent. However, in order to observe a more reliable trend, a systematic comparison of a much larger amount of data is required.

**Table 3:** Table showing different types of porous carbon materials used for hydrogen storage applications

Type of carbon	Modification	BET surface area/m^2^ g^−1^	Pore volume^a^ (0.95–0.99) (micropore volume)/cm^3^ g^−1^	Excess hydrogen capacity/wt% (temperature, pressure)	Comments	Reference, year
Graphene	Perforated graphene	∼2900	1.4	5.5 (77 K, 30 bar)	Perforated graphene by KOH activation	103 (2015)
	Graphene nanosheets	640	—	0.72 (298 K and 100 bar)	—	104 (2010)
	Hierarchical graphene	1305	—	4.01 (77 K, 1 bar)	—	105 (2013)
	Pd-Doped graphene sheet catalyst/activated carbon composite (Pd-GS/AC)	—	—	0.82 (298 K, 8 MPa)	—	82 (2013)
	1% Pd-doped graphene	—	—	8.67 (298 K, 60 bar)	—	106 (2016)
	Pt-Doped graphene	478.3	1.81	0.15 (303 K and 57 bar)	Hydrogen capacity increased by a factor of 2.23 compared to undoped graphene	107 (2011)
	Graphene oxide	—	—	1.4 (298 K and 50 bar)	—	108 (2012)
	Reduced graphene oxide–Mg nanocomposite	—	—	6.5 (473 K and 15 bar)	Recyclability tested up to five times	109 (2016)
	P-Doped graphene	288.4	(1.019)	3.66 (298 K and 4 MPa)	1.03 wt% capacity loss after four adsorption–desorption cycles at 298 K and 4 MPa	110 (2022)
	5% Pd-doped graphene	—	—	7.16 (298 K, 60 bar)	—	106 (2016)
	5% Ni-doped graphene	—	—	1.18 (298 K, 60 bar)	—	106 (2016)
	Ni/Pd alloy doped reduced graphene oxide (rGO)	—	—	2.65 (298 K, 4 MPa)	—	111 (2023)
	N-Doping of Pd decorated graphene	—	—	1.92 (298 K, 4.4 MPa)	Enhancement of hydrogen uptake capacity by almost 272%	112 (2012)
	Al-Doped graphene	—	—	5.13 (300 K and 0.1 GPa)	Computational	113 (2010)
	Palladium-phosphide-modified P-doped graphene	217.22	0.188	3.79 (298 K, 4 MPa)	Calcinated at 500 °C	114 (2023)
	Ni-B-rGO (nickel decorated boron doped reduced graphene oxide)	85	0.36 cc g^−1^	6.9 (77 K and 20 bar)	—	115 (2023)
	B-rGO (boron-doped reduced graphene oxide)	119	0.98 cc g^−1^	8.2 (77 K and 20 bar)	—	115 (2023)
Activated carbon	Nitrogen doped activated carbon	1646	0.70	2.14 (77 K and 0.93 bar)	—	116 (2023)
	Biomass-derived carbon (cigarette butt)	4300	2.09 *P*/*P*_0_ is 0.99	8.1 (77 K and 20 bar)	KOH activated	102 (2017)
	Activated carbon	2451	1.14	5.9 (77 K, 4 MPa)	—	117 (2011)
	Cellulose acetate derived AC	3771	1.75	7.0 (77 K and 20 bar)	KOH activation at 700 °C	53 (2017)
	Activated carbon (furfural)	2179	1.03	5.4 (77 K and 20 bar)	F-1/4-700	118 (2011)
	Activated carbon (glucose)	2121	1.00	5.3 (77 K and 20 bar)	G-1/4-700	118 (2011)
	Activated carbon (starch)	2194	1.01	5.6 (77 K and 20 bar)	S-1/4-700	118 (2011)
	Activated carbon (cellulose)	2370	1.08	5.6 (77 K and 20 bar)	C2-1/4-700	118 (2011)
	Activated carbon (eucalyptus sawdust)	2252	1.03	5.6 (77 K and 20 bar)	E1/4-700	118 (2011)
	Activated carbon monolith (ACM)	—	(1.04)	2.97 (77 K and 4 MPa)	Compressed monolith samples have slightly higher hydrogen storage than powder samples	119 (2008)
	AX-21_33	3363	—	57.1 mg g^−1^ (77 K and 2.5 MPa)	—	96 (2016)
	Polythiophene-derived activated carbon	3000	1.75	5.71 (77 K, 20 bar)	Chemically activated with KOH	120 (2011)
	Commercial AC (MSC30)	3305	1.596	5.78 (77 K and 4 MPa)	—	121 (2021)
	Commercial AC MSP20X	2363	0.935	4.74 (77 K and 4 MPa)	—	121 (2021)
	Commercial AC SA20	1737	1.190	3.24 (77 K and 4 MPa)	—	121 (2021)
	Commercial AC SA1500	2204	1.331	4.04 (77 K and 4 MPa)	—	121 (2021)
	Commercial AC TH90I	1204	0.495	2.90 (77 K and 4 MPa)	—	121 (2021)
	Commercial AC Filtrasorb400	983	0.469	2.34 (77 K and 3 MPa)	—	121 (2021)
	Biomass derived carbon (corn cob)	3012	1.7	2.0 (77 K, 1 atm and 0.6)	Chemically activated using KOH, K_2_CO_3_, or NaOH	122 (2010)
	Biomass derived carbon (corn cob)	3708	1.99	5.80 (77 K and 4 MPa)	—	123 (2014)
	Biomass-derived carbon (tamarind seeds)	1785	0.94	4.73 (298 K, 4 MPa)	KOH chemical activation	124 (2015)
	Biomass derived carbon (rice husk)	2537	1.31	1 (303 K, 25 MPa)	—	125 (2013)
	Biomass derived carbon (coffee bean waste)	2070	—	4.0 (77 K, 4 MPa)	Chemically activated using KOH	126 (2011)
	Pt-Doped activated carbon (AX-21)	2518	1.22	1.2 (298 K and 10 MPa)	5.6 wt% Pt doping	127 (2007)
	Biomass derived carbon (*Posidonia oceanica*)	2810	0.48	6.3 (77 K and 8 MPa)	—	128 (2020)
	Biomass derived carbon (wooden chips)	2835	0.73	6.4 (77 K and 8 MPa)	—	128 (2020)
	Biomass derived carbon (Meluca)	1806	0.82	2.16 (77 K and 1.0 MPa)	—	128 (2020)
	Biomass derived carbon (hemp stem)	3018	1.73	2.94 (77 K and 0.1 MPa)	KOH activation at 4.5 : 1 ratio at 800 °C for 1.5 hours	129 (2017)
	Pt-Doped biomass derived carbon (olive pomace)	1128	0.47	0.53 (298 K and 180 bar)	+1.10 wt% Pt	130 (2017)
	Horse-chestnut derived activated carbon	2270	—	4.01 (77 K and 20 bar)	ZnCl_2_ activating agent at 600 °C	131 (2024)
	Pd-Doped biomass derived carbon (olive pomace)	1074	0.45	2.46 (77 K and 40 bar)	+1.73 wt% Pd	130 (2017)
	C–Ni nanocomposite (agarose)	300	Total pore volume: 0.18 cc g^−1^	0.73 (298 K and 20 bar)	Pyrolysis at 400 °C	132 (2024)
	Biomass derived carbon (bagasse)	2580	1.27	2.62 (77 K, 1 bar)	KOH activation	133 (2020)
	Biomass derived carbon (cornstalk)	2464	1.21	2.61 (77 K, 1 bar)	KOH activation	133 (2020)
	Biomass derived carbon (pine powder)	2243	1.34	2.13 (77 K, 1 bar)	KOH activation	133 (2020)
	Pt-doped activated carbon (commercial AC)	—	—	0.9 (298 K and 10 MPa)	Hydrogen capacity increased three-fold when compared to undoped carbon	134 (2007)
	Pt/Pd doped activated carbon (corncob-derived)	2955	1.65	1.65 (298 K and 180 bar)	2.5% Pt and 2.5% Pd hybrid doped	135 (2014)
	Ni deposited on activated carbon	1957	1.090	1.6 (303 K and 5 MPa)	—	136 (2012)
	Biomass derived carbon (sucrose)	631.4	0.43	2.3 (123 K and 45 bar)	KOH : C mass ratio of 4 : 1	137 (2024)
	Biomass derived (saw dust)	3951	2.47	5.5 (77 K and 20 bar)	KOH activated at 800 °C	138 (2015)
	Biomass derived carbon (spider silk)	2730	1.56	3.7 (77 K and 25 bar)	—	139 (2023)
	Activated carbon (peanut shell)	3728.06	2.21	6.36 (77 K and 40 bar)	—	140 (2024)
	Activated carbon (beer lees)	2408	1.505	2.92 (77 K and 1 bar)	—	141 (2018)
	Activated carbon (coconut shell)	2800	2.17	0.85 (298 K, 100 bar)	—	142 (2007)
	Activated carbon (onion peel)	3150	1.64	3.67 (77 K, 1 bar)	—	143 (2020)
	Activated carbon (sword bean shell)	2838	1.54	2.63 (77 K, 1 bar)	KOH activation	144 (2019)
	Biomass derived carbon (*Polypodium vulgare* feedstock)	1234	0.57	2.73 (298 K, 45 bar)	—	145 (2024)
	Biomass derived carbon (walnut shells)	1163	0.491	2.6 (77 K, 1 bar)	—	146 (2024)
	Biomass derived carbon (pine tree bark)	351.45	—	0.73 (77 K and 90 bar)	LiCl was used as an activating agent at 900 °C	147 (2023)
Template derived carbons	Cr-MOF derived carbon	628	0.54	0.9 (77 K, 1 bar)	—	148 (2016)
	MOF-5 derived carbon	2393	1.13	2.7 (77 K, 1 bar)	—	148 (2016)
	Zeolite β derived carbon	3150	1.13	2.5 (77 K and 1 atm)	Chemical vapour deposition	101 (2007)
	Zeolite-templated carbon	3600	—	5.5 (77 K, 2.4 MPa)	—	149 (2012)
	ZIF-derived (Basolite Z1200) carbon	3188	1.94	6.2 (77 K and 20 bar)	After activation with KOH	150 (2012)
	Zeolite Y template derived carbon	2160	1.26	4.9 (77 K and under 20 bar)	—	151 (2010)
	Pt-Functionalised silica-templated carbon	712	0.84	1.1 (77 K and 1.8 MPa)	—	152 (2008)
	ZIF-templated carbon material	3323	2.32	6.5 (77 K and 20 bar)	Compaction under force of 5 tonnes	153 (2014)
	Ni-Doped silica templated carbon	560	—	2.2 (77 K and 10 bar)	—	154 (2015)
	Zn-Doped silica templated carbon	568	—	4.4 (77 K and 10 bar)	—	154 (2015)
	USY zeolite template using sucrose precursor	1200	—	3.65 (77 K and 20 bar)	—	155 (2014)
	Zeolite templated CNT	334	—	1.2 (308.15 K and 1 bar)	—	156 (2022)
	NH_4_Y zeolite template and furfuryl precursor	1886	0.866 (0.222)	0.92 (173.15 K and 1 bar)	Carbonization temperature of 750 °C & time of 3 h	157 (2013)
	Zeolite 13× template and furfuryl precursor	3332	1.66	6.2 (77 K and 2 bar)	Synthesis involves impregnation of furfuryl alcohol, then chemical vapour deposition (CVD) of ethylene at 700 °C	158 (2013)
	Zeolite EMC-2 template and acetonitrile precursor	3360	1.71	5.0 (77 K and 20 bar)	Prepared at temperature of 750 °C	159 (2011)
	Silica nanoparticles from rice husk as a template material and glycerol as carbon precursor	749	1.44	2.41 (77 K and 7.3 MPa)	Isosteric heat of adsorption 6.8 kJ mol^−1^	160 (2013)
	Silica nanoparticles from rice husk as a template material and sucrose as carbon precursor	644	0.85	1.42 (77 K and 1.9 MPa)	Isosteric heat of adsorption 7.3 kJ mol^−1^	160 (2013)
	Pt-Exchanged zeolite-templated carbons	2179	1.20	4.51 (77 K and 20 bar)	Acetonitrile as carbon precursor at 800 °C	161 (2011)
	Pt impregnated zeolite-templated carbons	2798	1.48	4.27 (20 bar at 77 K)	Acetonitrile as carbon precursor at 850 °C	161 (2011)
	Al-doped zeolite Y template carbon	1833	1.06	3.9 (77 K and 20 bar)	Acetonitrile precursor at 800 °C	162 (2011)
	Silica-derived carbon	1975	3.07	0.16 (173.15 K and 1 bar)	650 °C for 3 h	163 (2014)
	Co doped ZIF-67 templated carbon	1500	0.63	0.77 (30 bar and 300 K)	—	164 (2023)
	Pt-Doped zeolite EMC-2 templated carbon	2047	1.01	4.58 (77 K and 20 bar)	Prepared using acetonitrile precursor	165 (2020)
	Zeolite EMC-2 templated carbon	3360	1.71	6.33 (77 K and 20 bar)	—	165 (2020)
Carbon nanotubes	MWCNT	729.4	—	3.46 (298 K and 12.79 kPa)	—	166 (2021)
	SWCNT	—	—	2.91 (298 K, 0.174 MPa)	Molecular dynamics	167 (2024)
	Sulfur decorated SWCNT	764	0.78	1.70 (77 K and 9 MPa)	Endohedrally encapsulated sulfur	168 (2023)
	Fe@f-MWCNTs (carboxylate functionalised nanotubes)	18	—	0.55 (253 K, 70 bar)	Fe@f-MWCNTs prepared in DMF	169 (2017)
	Cu@f-MWCNTs (carboxylate functionalise nanotubes)	6	—	0.68 (253 K, 70 bar)	Cu@f-MWCNTs Prepared in DMF	169 (2017)
	MWNT with DyNi_2_ catalyst	—	—	3.3 (373 K and 94.7 bar)	—	170 (2008)
	Aryl-functionalised MWCNT	—	—	0.45 (77 K, 2.5 MPa)	—	171 (2021)
	SWCNT	700	—	0.8 (298 K, 3 MPa)	—	172 (2010)
	MWCNT	236.39	0.9563	0.2215 (77 K and 100 kPa)	Ball-milling at 700 rpm for 6 h	173 (2010)
	SnO_2_ MWCNT composites	—	—	2.03 (373 K, and 5 bar)	Ratio of MWCNT to SnO_2_ is 5 wt%	174 (2017)
	TiO_2_ decorated SWNT	—	—	0.45 (298 K and 30 atm)	—	175 (2014)
	Pd-SWNT	—	—	0.40 (298 K and 30 atm)	—	175 (2014)
	SWCNT	700	—	0.06 (298 K and 30 atm)	—	175 (2014)
	Ni/MWCNT	160	1.6331	2.27 (298 K and 8 MPa)	Synthesized by high energy ball milling for 8 h	176 (2014)
	Pt-MWCNT	160	—	2.9 (1.67 MPa and 298 K)	—	84 (2007)
	Pristine MWCNT	200	—	0.075 (1.67 MPa and 298 K)	—	84 (2007)
	Activated MWCNTs	479	1.073	0.54 (77 K and 1 bar)	Activated at 900 °C	177 (2012)
	HiPco™ single-walled carbon nanotubes (SWNTs)	—	—	0.43 (298 K and 8 MPa)	—	178 (2003)
	MWCNTs	729.4	—	3.46 (298 K and 12.79 kPa)	—	166 (2021)
	Boron and nitrogen co-doped CNT	87.7		1.96 (77 K and 16 bar)	Chemical vapor deposition synthesis	179 (2020)
	Li loaded activated MWCNT	174.19	0.19	1.33 (298 K and 21 bar)	—	180 (2020)
	Co loaded activated MWCNT	150.28	0.17	1.06 (298 K and 21 bar)	—	180 (2020)
	Activated MWCNT	359.18	0.27	0.82 (298 K and 21 bar)	MWCNT:KOH ratio of 1 : 5, 800 °C, and 1 h of activation	180 (2020)
	Pt doped MWNT	497	—	0.55 (77 K and 25 bar)		181 (2020)
	P-CNTs	—	—	0.65 (298 K and 80 bar)	—	182 (2018)
	F-CNTs	—	—	0.89 (298 K and 80 bar)	—	182 (2018)
	Pd-Functionalised CNT	—	—	1.7 (77 K and 1 atm)	—	81 (2012)
	PVP capped Pd-CNT	—	—	4 (77 K and 1 atm)	—	81 (2012)
	MWCNT	189	—	0.281 (77 K and 1 bar)	—	183 (2021)
	MWCNT-OH	153	—	0.321 (77 K and 1 bar)	—	183 (2021)
	MWCNT-O-Schiff base	119	—	0.370 (77 K and 1 bar)	—	183 (2021)
	MWCNT-O-Schiff base-Cu	116	—	0.396 (77 K and 1 bar)	—	183 (2021)
	Ni-MWCNTs	—	—	0.298 (298 K and 20 bar)	—	184 (2018)
	MWCNT	441.3		0.2 (298 K and 100 bar)		185 (2014)
	γ-Ray irradiated MWCNTs	—	—	1.2 (373.15 K and 1 atm)	150 kGy	186 (2017)

a Pore volume at *P*/*P*_0_ of ranges 0.95 to 0.99.

In the following sections, we will see that there exists great variability in reported hydrogen storage capacities of different types of carbon materials. Additionally, methods of improving the practical hydrogen uptake properties have been explored using various methods, such as by incorporating metals and other composite materials for enhancing thermodynamic properties. Table 3 summarises these approaches for various types of carbon materials like graphene, activated carbon, template derived carbons, carbon nanotubes and other types.

### 3.1. Graphene-based carbon materials

**Fig. 7:**
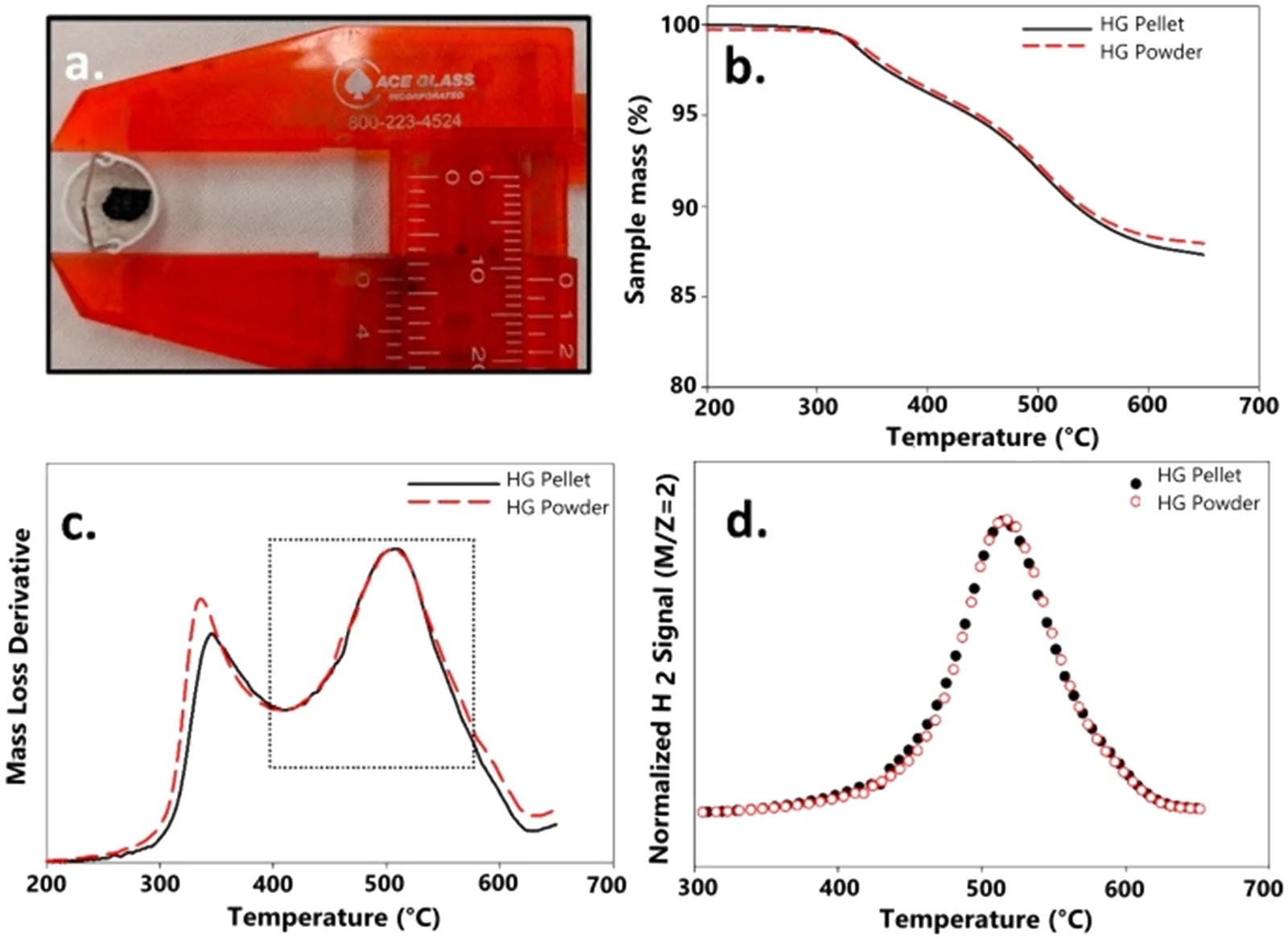
(a) Photograph of compressed hydrogenated graphite (HG) in a TGA pan. (b) TGA for HG pellets and HG powder. (c) Differential thermogravimetric analysis (DGA) for HG pellet and powder. (d) TGA-MS profile for *m*/*z* of 2 of HG pellets and powder samples. This figure has been reproduced from ref. 194 with permission from Elsevier, copyright 2021.

Graphene consists of a two-dimensional lattice of hexagonally arranged carbon, organised in a one or few-layered structures.^187^ Chemically, each carbon atom in graphene forms three covalent bonds with neighboring atoms, forming a 2-D plane of sp^2^ hybridized lattice. The perpendicular delocalized π-electrons along the plane govern the interactions between graphene layers, and give rise to its unique electrical properties, excellent thermal stability and mechanical strength.^188^ Theoretically, it has a surface area-to-mass ratio of 2630 m^2^ g^−1^ which makes it suitable for application of hydrogen storage.^189,190^ However, the actual surface area of graphene falls within a range of 150–600 m^2^ g^−1^ due to the tendency towards agglomeration of graphene layers.^191^ DFT calculations have demonstrated that van der Waals interactions between hydrogen and graphene play a major role in the adsorption mechanism.^192^ In addition, it has been shown that morphological factors like the curvature of graphene planes, defects, and inter-layer spacing greatly affect the hydrogen storage capacity of graphene.^191^ It was predicted by DFT calculations that the gravimetric capacity of graphene is optimal when the inter-layer spacing is between 0.6 nm and 0.8 nm.^193^ Hydrogenated graphene is one of the methods for hydrogen storage and was explored by various research groups, with a theoretical maximum capacity of 7.7 wt%.^194^ The main idea behind the hydrogenation of graphene, otherwise known as “graphane”, bypasses the limitation posed by the weak interaction of hydrogen with graphene *via* physisorption, by increasing the amount of hydrogen on the graphene surface by Birch reduction.^194,195^ Subrahmanyam *et al.* have explored this by post-synthetically modifying graphene *via* Birch reduction to form sp^3^ C–H bonds. These bonds can be broken upon heating or irradiation, leading to the release of chemisorbed hydrogen with a storage capacity of 5 wt%.^196^ In another study, Morse *et al.* have also investigated hydrogenated graphene (HG) showing a hydrogen storage capacity of 2.7 wt% and 2.8 wt%, for the pelletized and powder samples, respectively.^194^ As shown in Fig. 5, thermogravimetric analyses coupled with mass spectrometry (TGA-MS) analysis in this study (Fig. 7) indicate the evolution of hydrogen as a result of its thermal decomposition.

**Fig. 8:**
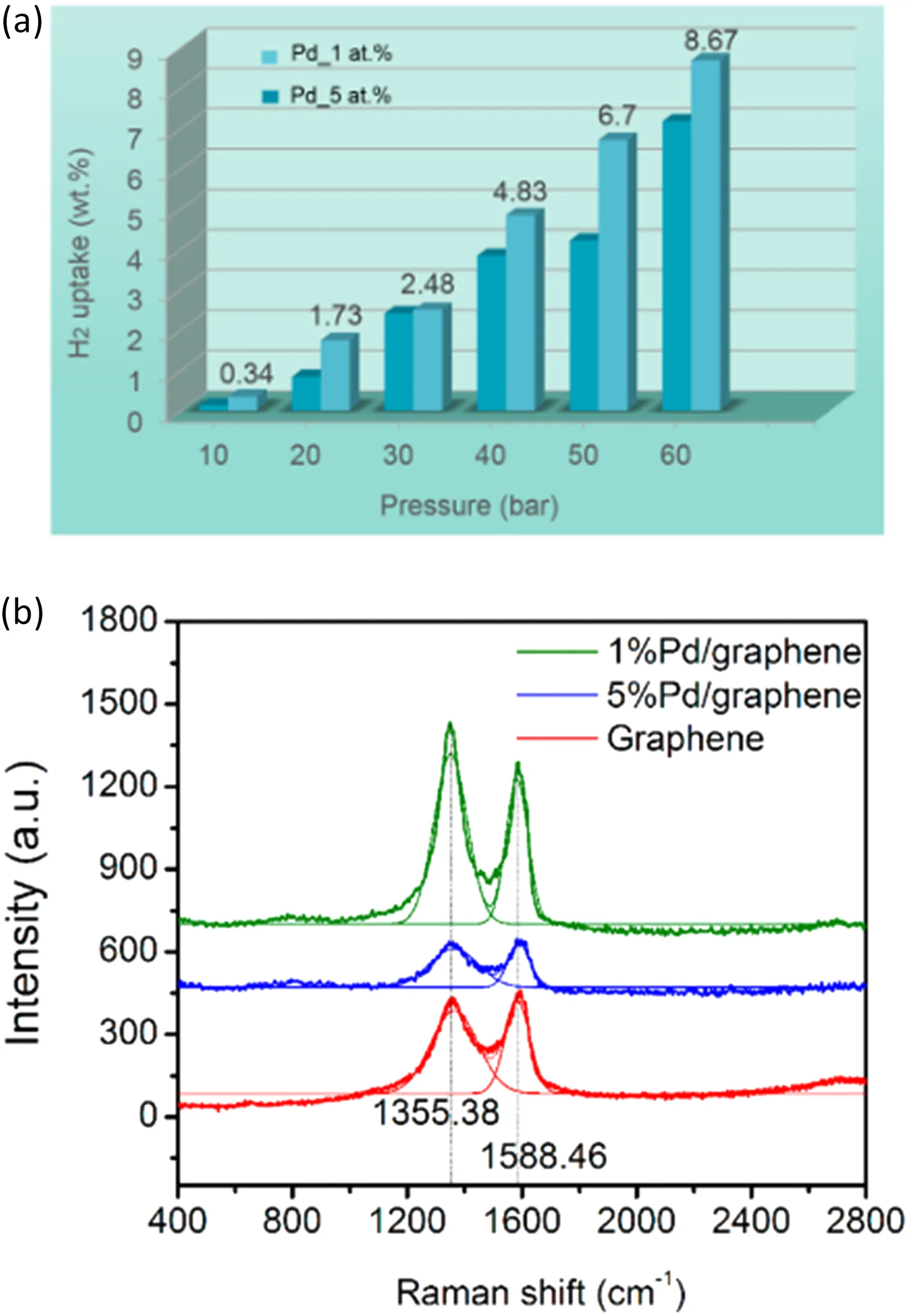
(a) Hydrogen uptake capacities of 1% Pd/graphene and 5% Pd/graphene functionalized graphene at various charging pressures. (b) Raman spectroscopy of graphene, 1% Pd/graphene, and 5% Pd/graphene, showing D and G bands at ∼1355.38 cm^−1^ and ∼1588.46 cm^−1^, respectively. This figure has been reproduced from ref. 106 with permission from American Chemical Society, copyright 2016.

Unmodified graphene usually has an uptake capacity of less than 1 wt% at ambient temperature and pressures ranging from 1 to 10 bar.^197^ Srinivas *et al.* have synthesised graphene nanosheets with a gravimetric storage of 0.1 wt% at ambient temperature and pressure of 10 bar.^104^ Moreover, the highest experimental H_2_ uptake capacity recorded for graphene was 3.1 wt% at 100 bar and 25 °C.^198^ Hence, although being a promising candidate material for hydrogen storage, on its own, graphene practically possesses very low hydrogen interactions to be used in practical applications. Various modifications have been explored to further enhance the hydrogen storage capacity of graphene which involves the decoration of graphene layer surfaces with various metals. Numerous reports have proved that dispersing alkali and transition metal nanoparticles greatly enhances the hydrogen uptake capacity by strengthening the binding of hydrogen to the material.^199^ This method utilizes a phenomenon known as the spillover effect, in which molecular hydrogen dissociates due to the presence of a catalyst, followed by the migration of hydrogen atoms onto the adjacent fragment of the substrate materials and their diffusion into the substrate surface.^200^ In a study, Zhou *et al.* have developed Pd-graphene composite sheets with a measured hydrogen uptake of 8.67 wt% at a pressure of 6 MPa and ambient temperature.^106^ As shown in Fig. 8a, the amount of Pd dispersed in the composite greatly affects the hydrogen storage capacity. This was attributed to the structural change caused by the amount of decorator metal on the graphene structure. As shown in Fig. 8b, the 1% Pd : graphene ratio resulted in a higher D/G band ratio in the Raman spectra, accounting for more disorder and resulting in higher hydrogen uptake. In addition to experimental data, theoretical calculations have also predicted incredible hydrogen storage capacity for metal-decorated graphene. *Ab initio* calculations by Ao *et al.* on Al-decorated graphene predicted an impressive hydrogen storage capacity of 13.79% under ambient conditions.^113^ Other computer analyses investigated storage capacities for Si, Li, and Ca decorated graphene.^201,202^

Heteroatom doping in metal-decorated graphene has opened up new avenues for achieving even higher hydrogen storage capacities. Heteroatom doping involves the introduction of atoms into the graphene chemical structure including either chemically substituting carbon with atoms like P, S, and N into the graphene lattice or the addition of halogen atoms through the formation of a C–X bond.^203^ Alongside metal nanoparticle decoration, both approaches can introduce defects into the graphene structure and allow for fine-tuning of the electronic and chemical properties of graphene, leading to synergistic effects, by (i) increasing the surface area for adsorption, (ii) introducing defects that would minimize nanoparticle agglomeration, and (iii) altering the charge density of the graphene surface, resulting in increased enthalpy for better hydrogen adsorption, all of which can significantly enhance hydrogen uptake capacity.^203–205^ Vinayan *et al.* have explored the fabrication of Pd functionalized N-doped solar exfoliated graphene (SG) composites.^206^ In their work, Pd/N-SG was found to have a higher hydrogen uptake capacity of 4.3 wt% at 25 °C and pressure of 4 MPa, compared to 0.68 wt%, 1.06 wt%, and 3.57 wt% for SG, N-SG, and Pd-SG, respectively. Moreover, the metal-decorated graphene composites displayed 90% adsorption of hydrogen at room temperature, demonstrating high reversibility. In another study the Pd_3_P decorated P-doped graphene composite demonstrated an uptake capacity of 3.66 wt% at 4 MPa and 25 °C, with effective 4 cycles of adsorption and desorption.^110^ DFT calculations have also investigated the surface functionalization of N-doped holey graphene with light metals like Na, K, and Mg,^207^ and estimated the highest loading capacity of 6.5 wt% and 5.5 wt% for Mg and Na functionalization, respectively. Other methods of utilizing graphene involve its use as a support in composites for other hydrogen storage materials. Cho *et al.* have explored incorporating N- and B-doped graphene for the nanoencapsulation of Mg nanoparticles.^208^ In their research, the graphene functionalized composites showed enhanced adsorption kinetics of hydrogen into Mg nanoparticles by the reduction of activation energy. Furthermore, introduction of heteroatom doping together with the existing structural defects of the graphene lattice aided in charge transfer, contributing to faster kinetics and higher hydrogen loading.^209^ Zhang *et al.* have synthesized fluorinated graphene composites of LiBH_4_,^210^ which showed superior desorption kinetics when compared to bare LiBH_4_, including lowering the desorption temperature by 120 °C and decreasing the activation energy of dehydrogenation from 180.10 kJ mol^−1^ to 130.87 kJ mol^−1^. In another study, researchers have used graphene nanoplatelet (GNP) structures that are composed of up to 100 graphene layers, along with Mg nanoparticles, as composites to solve the agglomeration issue of metal nanoparticles.^211^ The study has reported that by controlling the size of the GNP, the tendency towards agglomeration of Mg nanoparticles can be controlled, with smaller GNP particles resulting in higher agglomeration. As indicated from previous examples from the literature, the use of precious metals like Pd can greatly enhance the ambient storage capacity due to the possible spillover effect. However, on a practical basis, this can hinder the potential use of such composites due to material cost and poor abundance. Chen *et al.* have fabricated Ni/Pd alloy-doped graphene composites to reduce the dependence on Pd alone for doping.^111^ Through DFT calculations, the composites have shown a H_2_ storage capacity of 2.65 wt% at room temperature and pressure of 4 MPa.

**Fig. 9:**
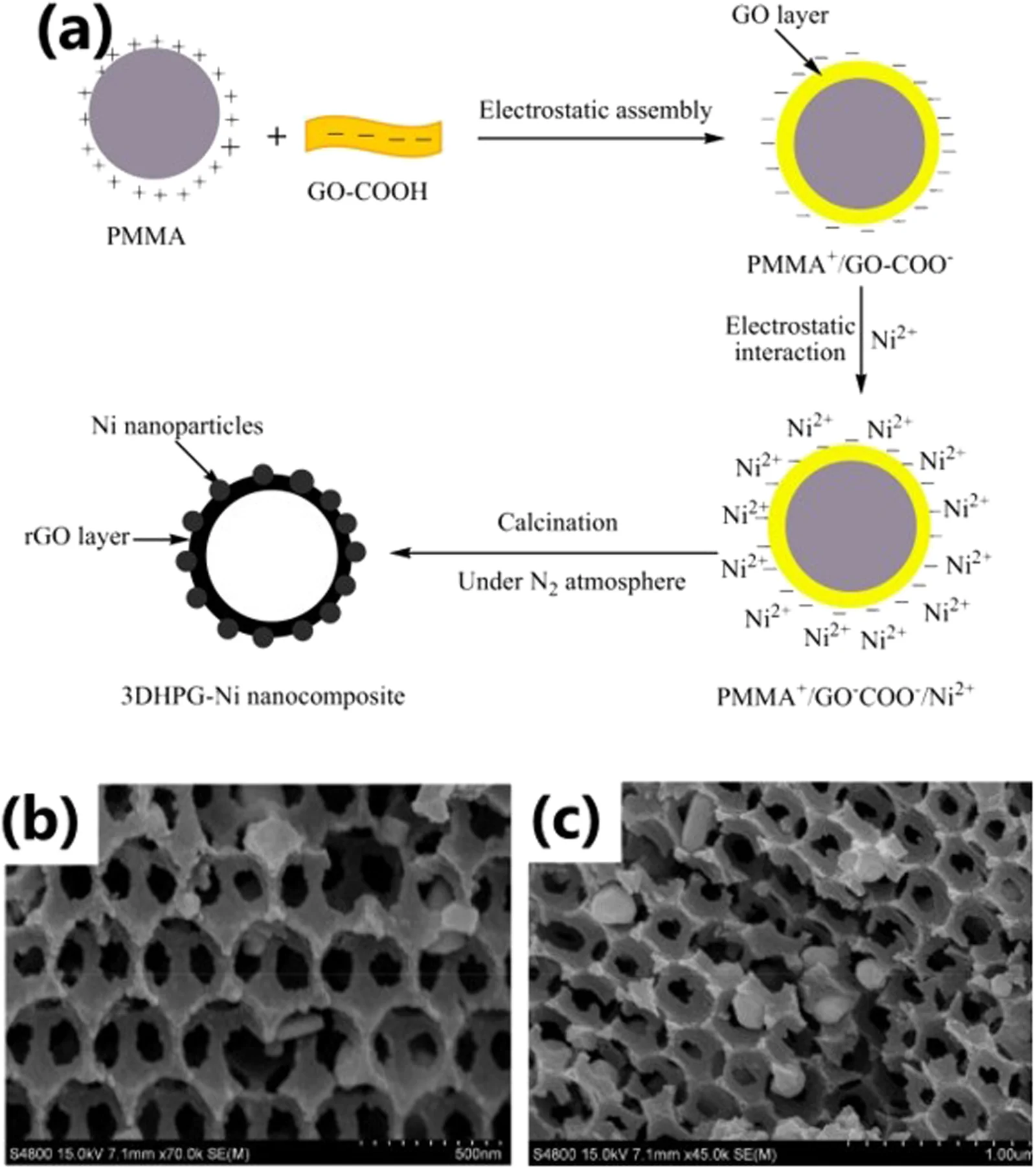
(a) Schematic illustrating the synthesis for three-dimensional porous hierarchical graphene. (b and c) SEM images of three-dimensional porous hierarchical graphene. This figure has been reproduced from ref. 213 with permission from Elsevier, copyright 2018.

Apart from monolayer graphene configurations, three-dimensional (3D) graphene materials are generating increasing interest as hydrogen storage materials. With an SSA that can reach as high as 3400 m^2^ g^−1^,^212^ 3-D graphene structures have an intricate interconnected network of graphene sheets which provides a high surface area for interactions with hydrogen. Liu *et al.* have synthesized three dimensional porous hierarchical graphene using polymethyl methacrylate (PMMA) microspheres as a template, by the deposition of negatively charged GO-COOH nanosheets on positively charged PMMA (Fig. 9).^213^ The nickel-functionalized 3D graphene exhibited a surface area of 925 m^2^ g^−1^ and a pore volume of 0.58 cm^3^ g^−1^ showing a maximum hydrogen capacity of 4.22 wt% and 1.95 wt% at 77 K and 25 °C at 0.5 MPa, respectively. In 2015, Klechikov *et al.* reported activated graphene scaffolds, with an ultra-high surface area of more than 3000 m^2^ g^−1^ and a pore volume of 2.2 cm^3^ g^−1^.^212^ The activation treatment using high temperature annealing of reduced graphene oxide (r-GO) resulted in the development of the 3D scaffold structure with high defects and interconnected layers, giving rise to an uptake capacity of 4.23 wt% of H_2_ at 12 MPa and 193 K, with a maximum saturation capacity of 7.48 wt% at 77 K. Jung *et al.* have explored using interconnected 3D graphene foams with modified porosity and Pt functionalization, achieving an uptake capacity of 3.19 wt% at 25 °C and a pressure of 10 MPa.^83^ Other studies have also explored hydrogen storage using graphene aerogels and MgH_2_ graphene aerogel (GA) composites.^214^

As can be seen from the above examples, both theoretical and experimental studies on the adsorptive capacities of graphene materials show its potential as a hydrogen storage material, when compared to other materials. Graphene can show relatively high surface areas and good uptake capacities when modified with not only precious metals like Pd, but also inexpensive dopants, such as Ca, Li, and Na.

### 3.2. Carbon nanotubes

**Fig. 10:**
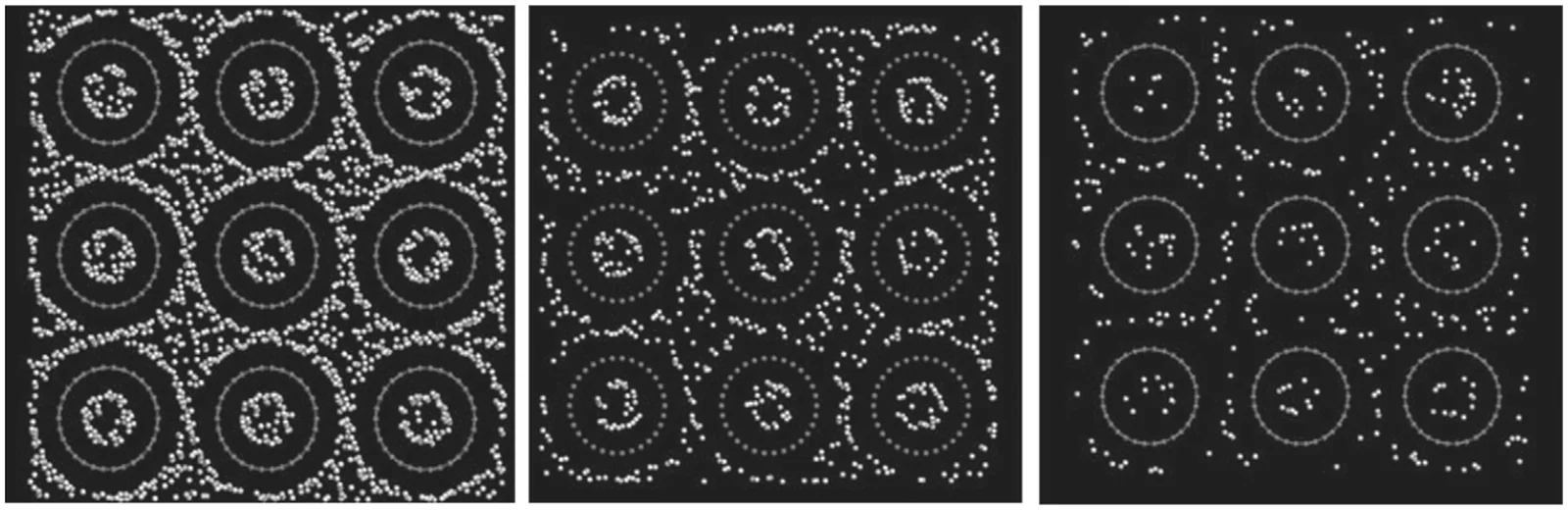
Grand canonical Monte Carlo simulations showing the sites of hydrogen molecules around carbon nanotube taken at conditions of 100 bar pressure at 77 K (left), 175 K (middle), and 293 K (right). This figure has been reproduced from ref. 219 with permission from Elsevier, copyright 2011.

Carbon nanotubes (CNT) are cylindrical structures of rolled graphene. There are various allotropes of CNTs, such as single-walled carbon nanotubes (SWCNTs) which are made from a single sheet of rolled graphene, and multi-walled carbon nanotubes (MWCNTs) which are formed as a result of multiple concentric layers of carbon nanotubes with interlayer distances being around 3.3 Å.^215^ CNTs have been reported with a wide range of surface areas from 50 to 1300 m^2^ g^−1^.^190^ In 1997, Dillon *et al.* demonstrated the potential of SWCNT materials for the reversible storage of hydrogen, showing an uptake capacity ranging from 5 to 10 wt% at ambient temperature and 0.04 MPa.^216^ Since then, carbon nanotubes have been extensively studied due to their unique morphology, high SSA, high tensile strength and thermal stability. However, discrepancies were reported in later studies for hydrogen storage capacities of CNTs, showing less than 1.7 wt% hydrogen storage capacity under 12 MPa pressure at room temperature.^217^ This variability among results might be dependent on external and internal factors involving the measurement techniques, devices, and synthesis methods.^218^ Furthermore, these inconsistencies highlighted the need for a better understanding of the mechanisms of hydrogen adsorption in CNTs. Both theoretical and experimental data suggest the involvement of physisorption and chemisorption as plausible adsorption mechanisms.^185^ However, despite the uncertainty surrounding the interaction of hydrogen with CNTs, it is typically regarded that the hydrogen molecules are adsorbed as a monolayer on the interior surface (endohedral), exterior surface (exohedral), or between the spaces left between bundles of CNTs.^219^ Additionally, like other carbon materials, chemical surface composition, surface area, and temperature greatly influence the hydrogen storage capacity. As shown in Fig. 10, grand canonical Monte Carlo (GCMC) simulations showed the effect of temperature on hydrogen adsorption capacity of carbon nanotubes, predicting higher hydrogen uptake capacity at lower operating temperatures.^219^

**Fig. 11:**
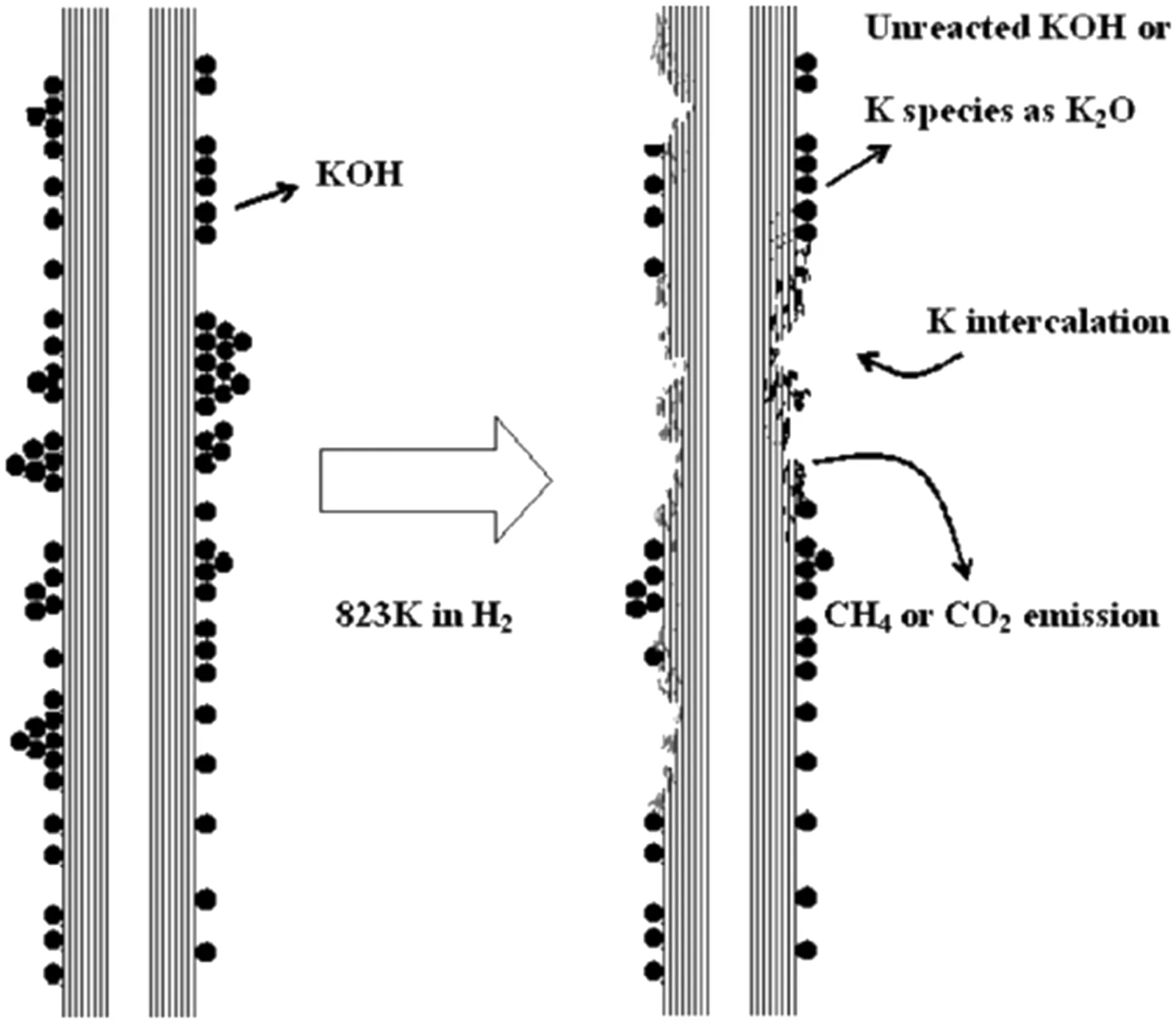
Schematic showing the effect of the activation process on the structural morphology of CNTs. This figure has been reproduced from ref. 220 with permission from Elsevier, copyright 2007.

Despite the challenges, various ways were investigated to improve the textural and chemical properties of CNTs to achieve higher H_2_ storage capacities.^218^ For example, modification of CNTs by chemical and physical activation showed enhanced porosity and introduced beneficial defects. As illustrated in Fig. 11, Chen *et al.* have shown that following KOH-treatment, MWCNTs showed a significant increase in H_2_ storage capacity from 0.71 to 4.47 wt% at ambient temperature and pressure of 0.1 MPa.^220^

Another form of modification reported involves the use of irradiation. For example, Silambarasan *et al.* have investigated the effect of γ-radiation on the structure of MWCNTs, and it was found that higher doses of around 100–150 kGy of γ-radiation are sufficient to show increased *I*_D_/*I*_G_ ratios in their Raman spectra indicating introduction of structural defects *via* the Compton effect, leading to an increased hydrogen storage capacity of up to 1.2 wt% at temperature of 100 °C and ambient pressure.^186^ Ball-milling is another way of introducing defects in the CNTs. The sheer and frictional forces introduce defects by shortening the length, increasing the surface area and increasing the amorphous nature (higher *I*_D_/*I*_G_), leading to improved hydrogen uptake.^173^ Liu *et al.* have obtained a six-fold increase in hydrogen storage capacity at ambient room temperature and pressure of about 8–9 MPa for MWCNTs when treated with ball milling for 10 hours.^221^

**Fig. 12:**
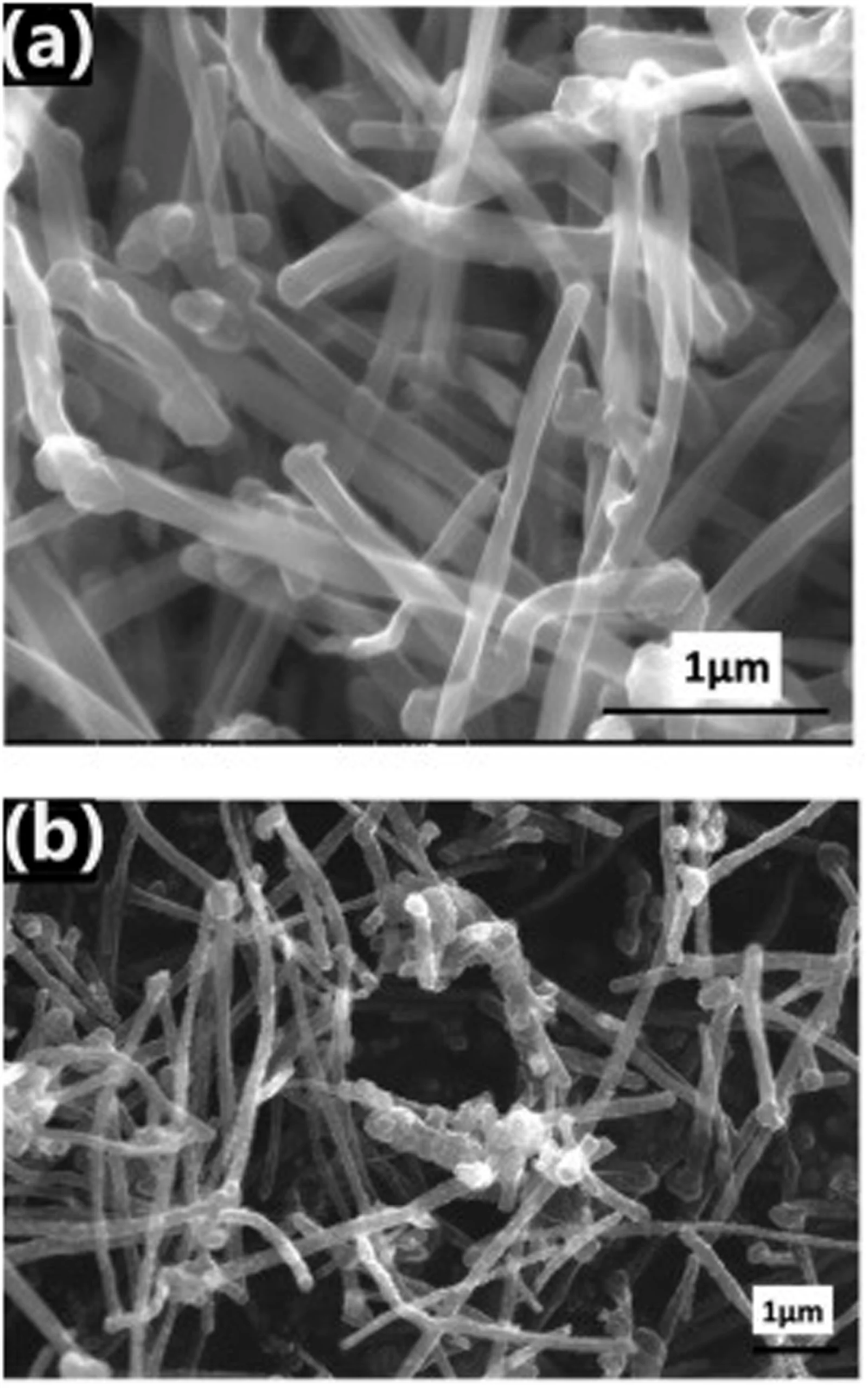
SEM images of (a) pristine MWCNTs and (b) MWCNT:SnO_2_ composites. This figure has been reproduced from ref. 174 with permission from Elsevier, copyright 2018.

In addition to physical methods that enhance the morphology of CNTs, metal-doping has also been used to improve hydrogen storage capacity and increase the hydrogen–adsorbate interaction. Like other carbon materials, doping with metals facilitates enhanced hydrogen adsorption.^222^ DFT calculations by Liu *et al.* on the effect of Li-doping of CNTs have found a high uptake capacity of 13.45 wt%, which can be attributed to Kubas binding.^223^ Other groups have also concluded higher storage capacity when compared to undoped MWCNTs at 0.075 wt% and 2.9 wt% for MWCNTs and Pt-MWCNTs, respectively.^84^ Doping of CNTs with metals like Cu, Mg, Ni, Ti and Al has been also reported.^169,218^ Nanoconfinement and incorporation into composites have also been explored for CNTs. For example, Khalilov *et al.* have investigated the nanoconfinement of transition metals in SWCNTs endohedrally through molecular dynamic (MD) simulations.^167^ The addition of Ni atoms to the inner structure of SWCNTs contributed to an increase of gravimetric uptake from 1.86 to 2.91 wt% when compared to unfunctionalized SWCNTs at ambient temperatures and pressures of 0.17 MPa. Other than transition metals, the hydrogen capacity of CNTs can be enhanced by endohedral doping with heteroatoms, like sulfur.^168^ Other types of CNT composites involve the incorporation of metal oxides. Vellingiri *et al.* have reported an uptake of 2.03 wt% at 100 °C and 5 bar through the use of SnO_2_ impregnated MWCNTs (Fig. 12).^174^ The addition of SnO_2_ contributed to the formation of defect sites. Additionally, the SnO_2_ particles can provide sites for catalytic activity that can facilitate hydrogen adsorption. A number of studies have concluded that hydrogen interacts with SnO_2_ and other metal oxide CNT composites *via* electrostatic and weak chemisorptive forces.^175,224^

Besides nanoparticles, CNTs can be used as support materials for polymer composites in applications of hydrogen storage, acting as support materials for polymers such as polyaniline, polyvinylpyrrolidone (PVP) and polypyrrole.^225,226^

Despite the discrepancies surrounding CNTs for hydrogen storage, studying this class of materials can provide significant understanding of hydrogen–carbon interactions. Additionally, they exhibit the potential to be used alongside other materials as composites for modification of textural, physical, and chemical properties for hydrogen storage materials. In general, MWCNTs and SWCNT have shown an uptake capacity of up to 3.5 wt% at pressures up to 140 bar.^201^

In terms of adsorptive capacity, it is thought that carbon nanotubes offer no practical advantages when compared with other porous materials like MOFs and activated carbon, due to weak thermodynamics.^74^ The average enthalpy of adsorption for SWCNTs of diameter 0.8 nm is 8–9 kJ mol^−1^, decreasing to 5.9 kJ mol^−1^ with a larger diameter of 1.2 nm.^227^ As highlighted by Cheng *et al.*,^228^ the successful use of carbon nanotubes for practical hydrogen storage requires (i) finding methods of mass production of CNTs, (ii) development of effective pretreatment and modification methods for improving hydrogen storage, (iii) studying the cyclability and kinetic behaviour of adsorption of hydrogen, and (iv) use of theoretical interpretation to guide the pathway for developing CNTs.

### 3.3. Activated carbon

Unlike the ordered structure of graphene and CNTs, activated carbons (ACs) are made up of intricate pore networks of graphite crystallites and amorphous carbon. These disordered structures give rise to ultra-high surface areas often exceeding 3000 m^2^ g^−1^, slit-shaped pore morphology, and a variety of pore volumes ranging from the macro, meso, to microporous range.^229^ ACs have been reported to show the highest adsorption storage capacities reported to date among the different types of carbon materials between 77 K and 300 K.^230,231^ What makes this class of carbon materials unique as hydrogen storage materials is their availability and tunable properties; ACs can be synthesized from the pyrolysis and activation of precursors from a wide range of carbon-rich materials either from natural (biomass derived) or unnatural sources, including coal, petrochemical materials, peat, polymeric materials, and various agricultural derivatives like wood, bamboo, and food waste.^232^ Lignocellulosic biomass is one of the most popular precursors for synthesizing activated carbon. Most of the carbon in biomass materials exists in different forms, including lignin, hemicellulose, and cellulose, and their composition in the initial biomass precursor affects the final morphology of the activated carbon, including its surface area and micropore volume.^233^ Pyrolysis is the thermal decomposition of precursor materials into carbon in the absence of oxygen.^234^ Besides pyrolysis, ACs can be activated *via* both physical and chemical methods to help enhance the porous and textural properties for better hydrogen storage. Physical activation involves high temperature gasification of the carbon material with an oxidizing gas (usually CO_2_ or H_2_O). Meanwhile for chemical activation, the raw material is treated first with an oxidizing agent at high temperature. Several chemical activation agents (occasionally called porogens), such as KOH, ZnCl_2_, H_2_SO_4_, H_3_PO_4_, and NaOH, are usually used. Studies have proved chemical activation to be more efficient, yielding a higher surface area and optimal microporosity.^48,235^ Together, pyrolysis and activation temperature and conditions were shown to play crucial roles in establishing ultra-high surface areas and desirable micro porosity.^236^ For example, in a study, the effect of carbonization temperature and activation conditions on lignin-derived activated carbon derived from a feedstock precursor using a physical activation method was explored.^237^ The use of higher carbonization temperatures was shown to result in reduced BET surface areas, as the porous network of micropores is further widened by the activation process, leading to the formation of larger micropores, mesopores, resulting in an overall decrease in surface area. Hence, lower carbonization temperature can sometimes achieve maximal microspore volumes and limit the pore widening effect of activation. The results from optimization of conditions yielded a surface area of 1409 m^2^ g^−1^, with a total H_2_ uptake of 1.9 wt% at 77 K and 0.1 MPa, using a low carbonization temperature to retain micropores, followed by a high activation temperature of 350 °C and 1000 °C, respectively. Similarly, this trend was also observed in other studies as activation was shown to develop and enhance the pore structures in coal-derived activated carbon.^238^

It was shown that in addition to porosity, other factors like surface morphology, composition and functionalization are important in determining the adsorptive capacity of AC. Exceptional 8.1 wt% excess uptake and 9.4 wt% total hydrogen uptake at 77 K and 20 bar pressure were achieved using AC derived from cigarette butts.^102^ The AC samples were synthesized by hydrothermal carbonization, followed by chemical activation with KOH, resulting in an ultra-high surface area of 4300 m^2^ g^−1^, 90% of which is a result of micropores. It was concluded *via* elemental analysis that the activation process yielded high oxygen content in the final AC product, mainly in the form of –OOH, –C–OH and HO–C=O functional groups. The surface composition of the AC contributed greatly in enhancing the hydrogen uptake capacity, as a result of the high binding energy between hydrogen and the oxygen-containing functional groups. In another study, KOH activated carbon derived from a bamboo precursor achieved a surface area of 3208 m^2^ g^−1^ and a micropore composition of 1.01 cm^3^ g^−1^.^239^ The maximum hydrogen uptake capacities at 77 K were 6.6 wt% at 40 bar and 2.74 wt% at 1 bar, respectively.

**Fig. 13:**
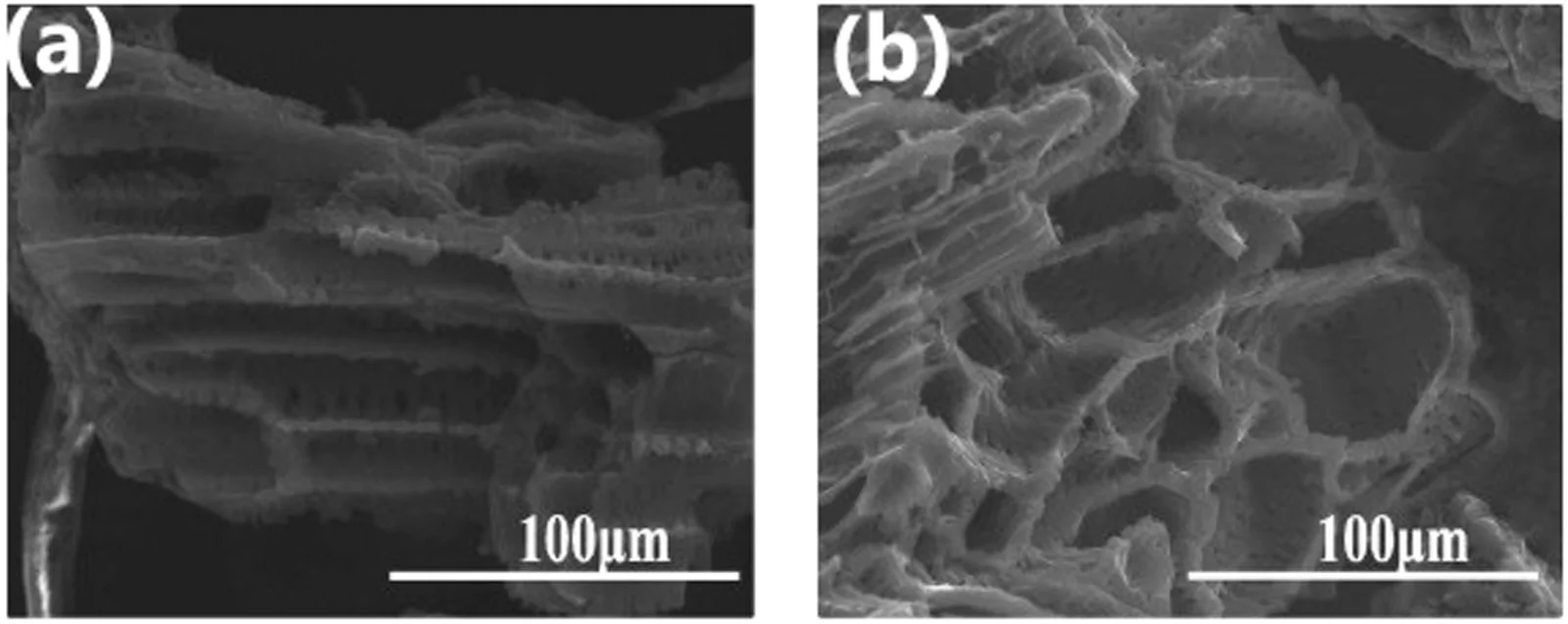
(a and b) SEM images of activated carbon derived from corncob using various chemical activation methods. This figure has been reproduced from ref. 122 with permission from Elsevier, copyright 2010.

Sun *et al.* have used corncob to synthesise porous carbon (Fig. 13) which was studied using different activation methods including two-step KOH activation with ultrasonication treatment. The obtained activated carbon showed a surface area of 3012 m^2^ g^−1^ and a pore volume of 1.7 cm^3^ g^−1^. Hydrogen adsorption studies showed an uptake of 2.0 wt% at a temperature and pressure of 77 K and 1 bar, respectively.

**Fig. 14:**
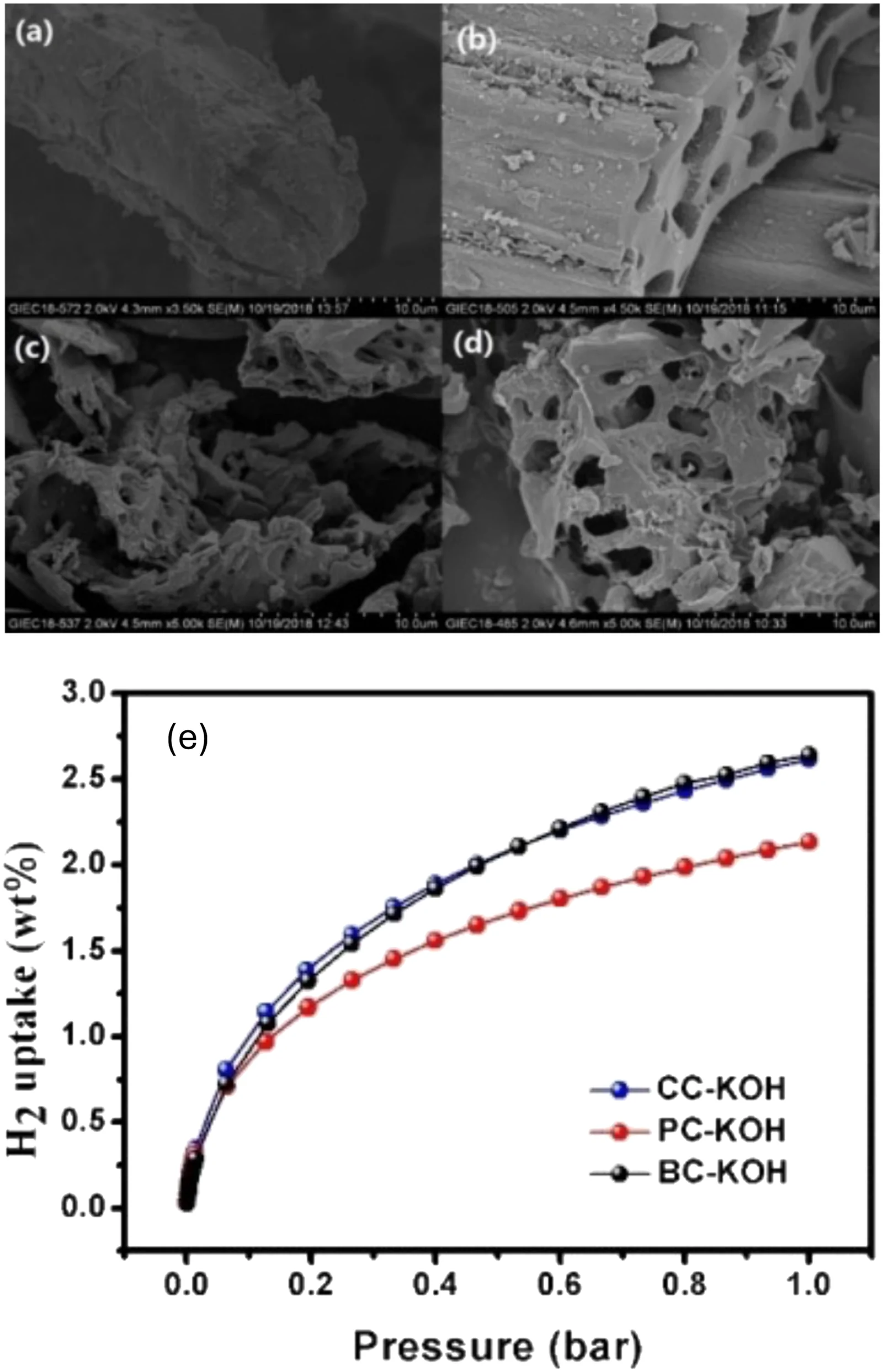
SEM image of carbon derived from cornstalk (CC), after (a) thermal activation at 800 °C, (b) ZnCl_2_ activation, (c) H_3_PO_4_ activation and (d) KOH activation. (e) H_2_ adsorption for the KOH activated carbons derived from bagasse, cornstalk and pine powder shown. This figure has been reproduced from ref. 133 with permission from John Wiley and Sons, copyright 2020.

Other studies also have explored different biomass sources like tamarind seeds, olive pomace, and coffee bean wastes. Activated carbons from fungi-based chars were also explored, with an achieved surface areas of 1600–2500 m^2^ g^−1^ and hydrogen storage capacity of 2.4 wt% at 1 bar and −196 °C and a saturated uptake ranging from 4.2–4.7 wt% at −196 °C and 35 bar.^240^ Peng *et al.* have explored different methods of chemical and physical activation of porous carbon derived from common biomass waste precursors like bagasse, cornstalk and pine powder.^133^ The resulting carbons were activated using CO_2_, ZnO_2_, and KOH. As shown with other literature reports, KOH was shown to have the best effect in developing a high micropore volume and surface area. Hydrogen adsorption studies have shown an uptake capacity of 2.62 wt%, 2.61 wt% and 2.13 wt%, for bagasse, cornstalk, and pine powder carbons, respectively. Additionally, as shown in Fig. 14, the adsorption isotherm showed type I adsorption indicating that the formed hydrogen monolayer reaches saturation at higher pressure, a typical behaviour of microporous materials.

Functionalization of AC results in enhanced hydrogen storage capacity and improved kinetics.^241^ A number of studies have investigated doping AC with metals like Pt, Cu, Ni and Pd to enhance hydrogen uptake capacity *via* the spillover effect.^134–136,242^

Flamina *et al.* have synthesised Ni-functionalised carbon nanocomposites with enhanced hydrogen storage.^132^ The composites were synthesised by preparation of aerogel by freeze drying a mixture of agarose and nickel acetate salt. The freeze-dried composites were then pyrolyzed at temperatures of 400, 600 and 800 °C to prepare Ni–C nanocomposites of Ni particles and nanotextured carbon. The carbon composite synthesised at 400 °C had a BET surface area of 300 m^2^ g^−1^, which is lower than the non-functionalised carbon. Despite the lower surface area, the composite showed 6.5 times more hydrogen storage capacity of 0.73 wt% at 298 K and 20 bar, which was attributed to the spill-over effect.

### 3.4. Template-derived carbon

**Fig. 15:**
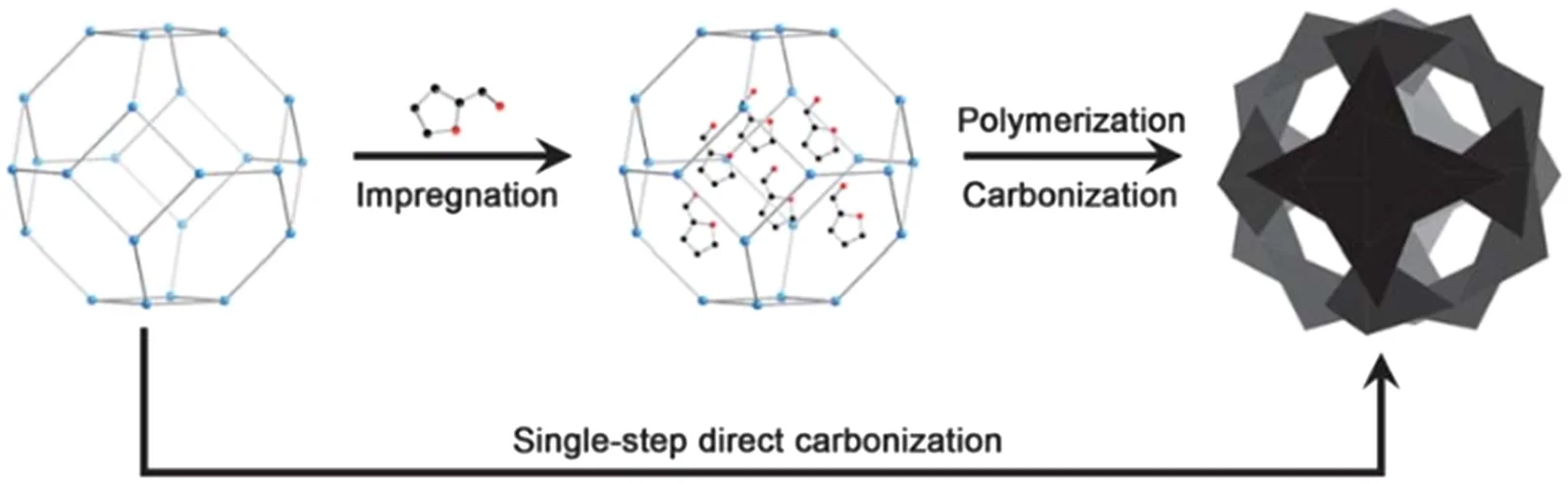
Scheme showing the steps of hard-template synthesis of templated carbon. This figure has been reproduced from ref. 245, with permission from Royal Society of Chemistry, copyright 2013.

Template-derived carbon is produced as a result of the pyrolysis of a template precursor. Unlike activated carbon, template derived carbons often have controlled hierarchical pore morphology and narrow pore size distribution depending on the synthesis method and precursors used. Template derived carbons can be synthesized *via* hard templating and soft templating methods. An example of hard-templating is shown in Fig. 15 involving the impregnation of a sacrificial template with carbon rich molecules by chemical vapor deposition (CVD) followed by carbonization. This results in a porous 3D negative structure of the template. Hossain *et al.* and others have used sucrose as a carbon source for synthesizing porous carbon,^137,155,243^ and several studies have explored the use of furfuryl alcohols.^158,244^

For example, choosing a silica-based template resulted in the formation of mesoporous carbon, while carbonization of zeolites formed porous carbon with a higher micropore volume showing better hydrogen storage capacity.^246^ Hydrogen adsorption capacities in template-derived carbon are among the highest reported to date for various types of carbon materials. For example, zeolite 13× derived carbon with a surface area of 3332 m^2^ g^−1^ and a pore volume of 1.66 cm^3^ g^−1^, respectively, showed a hydrogen uptake of 7.3 wt% at 20 bar and 77 K, with an estimated maximum uptake capacity of 9.22 wt%.^158^ Zeolite-β derived carbon materials with a surface area of 3200 m^2^ g^−1^ and a pore volume of up to 2.41 cm^3^ g^−1^ showed a hydrogen adsorption capacity of 6.9 wt% and a projected maximum of 8.33 wt% at 77 K and 2 MPa.^101^ A high hydrogen adsorption capacity of 5.5 wt% at 77 K and 30 MPa was achieved *via* zeolite templated-carbon (ZTC), with the surface area reaching 3600 m^2^ g^−1^.^149^ These high hydrogen uptake capacities are attributed to the higher micropore volume of zeolite-derived carbons.

**Fig. 16:**
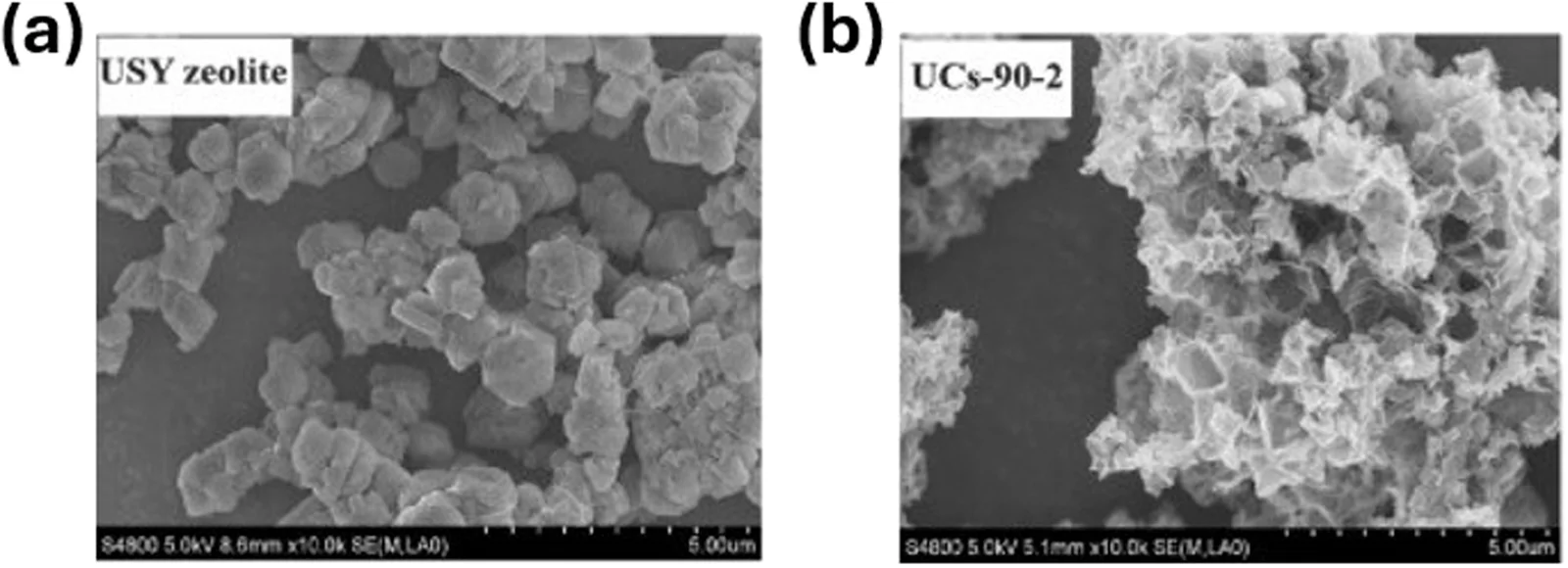
SEM images of (a) USY zeolite and (b) template derived carbon UCs-90-2. This figure has been reproduced from ref. 155 with permission from Elsevier, copyright 2014.

Cai *et al.* have used sucrose as a carbon precursor on an ultra-stable Y (USY) zeolite template shown in Fig. 16.^155^ The powdery zeolite was first mixed with sucrose at a ratio of 1 : 1 in an aqueous solution along with H_2_SO_4_. After a series of mixing and heating up to 160 °C, the resulting composite mixture was then heated under N_2_ at a heating rate of 5 °C min^−1^ up to until 900 °C for four hours resulting in the porous carbon (UCs-90-2) shown in Fig. 14. The template derived carbon showed a BET surface area of 1219 m^2^ g^−1^ and a total pore volume value of 1.068 cm^3^ g^−1^ with a hydrogen uptake capacity of 3.65 wt% at 77 K and 20 bar.

As for carbons derived from silica-based templates, several materials have been investigated for potential for storage of hydrogen, yielding mesoporous carbon that showed relatively low hydrogen storage capacity.^160,163^

Another template source for carbon involves the use of MOFs as precursors. MOFs are a class of crystalline three-dimensional structures of metal nodes interconnected by organic ligands, forming potential voids.^247^ Due to their high microporosity, with surface areas reaching up to ∼15 000 m^2^ g^−1^, MOF-derived carbons have been widely studied for hydrogen storage. Zn-based MOFs, IRMOF-1, IRMOF-3, and IRMOF-8 were used for the synthesis of MOF-derived carbon (MDC).^248^ Carbon derived from IRMOF-1 with a terephthalic acid linker resulted in the highest BET surface area of 3174 m^2^ g^−1^ and high ultra-microporosity when compared to 1678 and 1978 m^2^ g^−1^ for IRMOF-3 and IRMOF-8, respectively. Hydrogen uptake for IRMOF-1 derived carbon reached an exceptional 3.25 wt% at 77 K and 0.1 MPa. For MOF-derived carbons, the aromatic nature of the linker provides a good source of carbon, in addition, it has been shown that the metal-to-linker ratio affects the extent of porosity and surface area.^249^ A positive correlation between porosity and metal-to-linker ratio was observed for Zn-based MOFs; after carbonization, a Zn/C ratio equal to 0.07 and 0.25 yielded a surface area of 900 m^2^ g^−1^ and 1800 m^2^ g^−1^, respectively. Meanwhile an opposite trend was seen in a Cr-based MOF due to the possible formation of Cr-oxides and carbides after pyrolysis.^148^ Zeolitic imidazolate frameworks (ZIF) are another subgroup of MOFs that have imidazolate linkers. Mokaya *et al.* have synthesised ZIF-derived carbon from Basolite Z1200. The resulting carbon after activation had a surface area of up to 3200 m^2^ g^−1^ and a pore volume of 1.94 cm^3^ g^−1^, and an excellent hydrogen uptake capacity of 6.2 wt% at 2 MPa and 77 K was observed.^150^ Jiang *et al.* have also worked on using ZIF-8 as a template together with furfuryl alcohol as a precursor for synthesizing porous carbon, showing a BET surface area of 3405 m^2^ g^−1^ and a pore volume of 2.58 cm^3^ g^−1^ with a hydrogen uptake of 2.77 wt% at 77 K and 1 bar.^244^

Other forms of template derived carbon include carbide-derived carbons (CDC) which are synthesised by removing the metallic component from the carbide structure by chlorination to yield the carbon material with a uniquely narrow PSD and high microporosity. KOH activated porous carbon derived from Zr–C has shown a surface area of up to 2800 m^2^ g^−1^ and a hydrogen uptake of 6.2 wt% at 77 K and 2 MPa. Additionally, at 1 bar, activated CDC have shown an uptake of 2.7 wt% of hydrogen, which is one of the highest values to be recorded for porous carbon materials at this pressure.^250^ Other forms of precursors have also been investigated for microporous carbon structures, including polynuclear complexes.^251,252^

### 3.5. Other carbon structures

Besides the different types of carbon mentioned above, more miscellaneous forms of carbon have been explored due to their unique morphology and properties. Carbon nanospheres are hollow nanostructures of carbon that possess a high surface area-to-volume ratio, additionally, their hollow morphologies make them good candidates for nanoencapsulation. Carbon nanospheres have been investigated for hydrogen storage by Baca *et al.* at 313 K and a pressure of 4.5 MPa, and Pt-doped carbon nanospheres showed a hydrogen uptake of 0.48 wt%.^253^ In another study, CO_2_ activated nanospheres derived from starch showed a high surface area and pore volume of 3350 m^2^ g^−1^ and 1.75 cm^3^ g^−1^, respectively. The nanospheres showed a high hydrogen uptake of 6.4 wt% at 77 K and pressure of 2 MPa.^254^

Carbon nanorods are elongated carbon structures that are the by-product of CNT synthesis. Studies have explored their potential for hydrogen storage through temperature programmed reduction (TPR) measurements showing an uptake of 0.14 wt%.^255^ Carbon nanofibers (CNFs) are an allotrope of carbon synthesised *via* the deposition of gaseous hydrocarbons like methane and acetylene onto a metal catalyst. This creates carbon structures with varied morphology depending on the shape of the catalyst used, including herringbone, tubular morphology, and card deck (stacked). In 1998, Chambers *et al.* reported the synthesis and characterisation of graphite carbon nanofibers with herringbone arrangement and a questionably high hydrogen uptake of about 67 wt%.^256^ However, no one has been able to replicate these results successfully. Fullerenes are a group of peculiarly shaped nanostructures composed of hexagonal and pentagonal carbon meshes.^257^ Fullerenes can have ellipsoidal, spherical and tube-like shapes. However, the most stable structure of fullerene is C_60_, otherwise known as Buckminsterfullerene or buckyballs.^258^ Fullerenes have been studied *via* computer simulations for their use as hydrogen adsorbents. Scandium bound fullerenes have been investigated using DFT calculations with an estimated maximum hydrogen storage capacity of 9 wt%.^259^ Other studies have calculated a maximum hydrogen storage capacity of 8 wt% for Ti-decorated C_60_ under ambient conditions.^260^ Despite the high theoretical uptake capacity, most of these materials are yet to be tested experimentally. In the previous sections, we have highlighted widely studied types of porous carbon for hydrogen storage application. Studies on graphene have led to providing crucial insight in understanding H_2_–adsorbate interactions, and have shown an adsorption capacity of up to 3 wt%. Graphene can also be modified into 3D structures including aerogels and foams that can be incorporated into composites. Templated carbon has the advantage of high tunability and high surface area. While activated carbon can be produced from waste biomass pyrolysis and can achieve high surface areas and storage capacities, they offer less tunability than templated carbon. Each type of carbon and their preparation methods offer a range of advantages and disadvantages for hydrogen storage applications.

## 4. Life cycle assessment of carbon materials

While porous carbon materials can offer promising applications across various industries including the hydrogen energy sector due to their exceptional properties, their production and use come with significant environmental challenges. Life cycle assessment (LCA) is a tool that can be used for assessing the environmental impact of products to allow their development and optimisation, while encompassing factors like cost and environmental load such as waste and emissions along the life cycle of a product.^261^ The aim of LCA is to (i) reduce the consumption of non-renewable resources, (ii) decrease the emission and the release of harmful waste products, (iii) improve the product's efficiency, and (iv) improve the fate of the product at end-of-life (EOL) and its potential for reusability and recyclability.

As for CNTs, optimistic projections show an annual production of 7000 tons by 2025,^262^ with a composition of 20% single-walled carbon nanotubes (SWCNTs) and 80% multi-walled carbon nanotubes (MWCNTs), respectively. The materials needed for CNT synthesis required a carbon precursor which may be solid or gaseous alongside a metal catalyst. Additionally, various synthesis methods exist, including electric arc discharge, laser ablation, chemical vapor deposition (CVD), and high-pressure carbon monoxide (HiPco). The most common and oldest method to synthesise CNTs is electric arc discharge, where electric arc vaporises a hollow graphite anode packed with a mixture of transition metals and graphite powder, along with inert gas flow. Flowrate, gas pressure and metal concentration can be optimised to obtain a high yield of CNTs of up to 70%.^263^ CVD is another common method for CNT synthesis that produces a higher yield of CNTs (95–99%), with greenhouse gas emissions (GHG) ranging from 28.55 to 0.02 kg of CO_2_ emitted per one gram of material synthesised.^264^ Economic assessment done by Isaacs *et al.* has calculated the manufacturing costs per 1 g of SWCNT for arc discharge, CVD, and HiPco processes to be $1906 g^−1^, $1706 g^−1^, and $485 g^−1^, respectively.^265^ In addition to great variability in synthesis methods, cost and emissions produced, most methods of CNT synthesis are energy intensive. For example, the manufacturing and production of CNTs requires from 2 to 100 times more energy than materials like aluminium.^266^ Moreover, CNT production emits 10 000 times more GHG than other carbonaceous materials like graphite.^267^

Additionally, the environmental impact of CNT manufacture and production is substantial. LCA primarily associates CNT production to climate change, acidification, and depletion of inorganic resources.^268^ Gavankat *et al.* have analysed the environmental impact of a commercial SWCNT plant showing a high global warming potential of 153 and 818 kg of CO_2_ emitted per gram of CNT, synthesised by the fluidized bed CVD and CoMoCAT method, respectively.^269^ As for graphene carbons, the source of carbon material and the method of synthesis greatly impact their cost and environmental impact. It was calculated that using conventional methods, a kilogram of graphene oxide costs around $50–990,^270^ whereas synthesising graphene using flash-joule technology from biomass waste as a source of carbon can reduce its cost to around $5.41–8.84 per kg, in addition to mitigating the environmental impact resulting from the production of graphene by 10 fold when compared to conventional production methods.^271^

Similarly, activated carbon showcases varying potential for environmental improvement, with its processing location and choice of feedstock influencing global warming impacts and significantly affecting environmental footprints.^272,273^ Global warming impact can be reduced by 60% when the material is processed in a country with a low-carbon electricity system.^272^

As for synthesis steps, LCA done on activated carbon derived from biomass suggests that recycling of KOH involved in the activation process could reduce environmental emissions, lowering greenhouse gas (GHG) emissions to 83.76% as well as reducing production cost.^274^ Additionally, a pre-carbonisation step has a higher environmental impact than single-step activation.^275^

Although LCA provides a valuable framework for evaluating and mitigating environmental impacts, guiding the development of sustainable practices in the synthesis, manufacturing and production, the field of LCA for nanomaterials is still at its infancy and has many challenges. Most studies lack end-of-life analysis, while others focus only on the cradle to grave approach. Hischier and Walser have identified key issues, including defining the functional unit and impact assessment values and have suggested strategies to improve LCA studies by involving the collection of inventory data, addressing emissions, and establishing toxicity factors while properly defining the goal and scope of LCA studies.^276^

LCA of adsorbents for hydrogen storage application displays the environmental and economic implications of various materials used in storage systems, emphasizing the need for sustainable choices. In addition to environmental impact, hydrogen economy involves the integration of multiple sectors including heating, electrical and transportation.^277^ Besides material discovery and optimisation, energy and mass transfer processes should be also considered throughout the system.^278,279^ In addition, for viable and sustainable implementation, production of hydrogen from low-carbon and renewable resources like water electrolysis and photocatalysis,^280–282^ and biogas sources,^283^ as well as exergy analysis to evaluate the usable amount of energy must be considered.^284,285^

## 5. Challenges, current trend, and future outlook

**Fig. 17:**
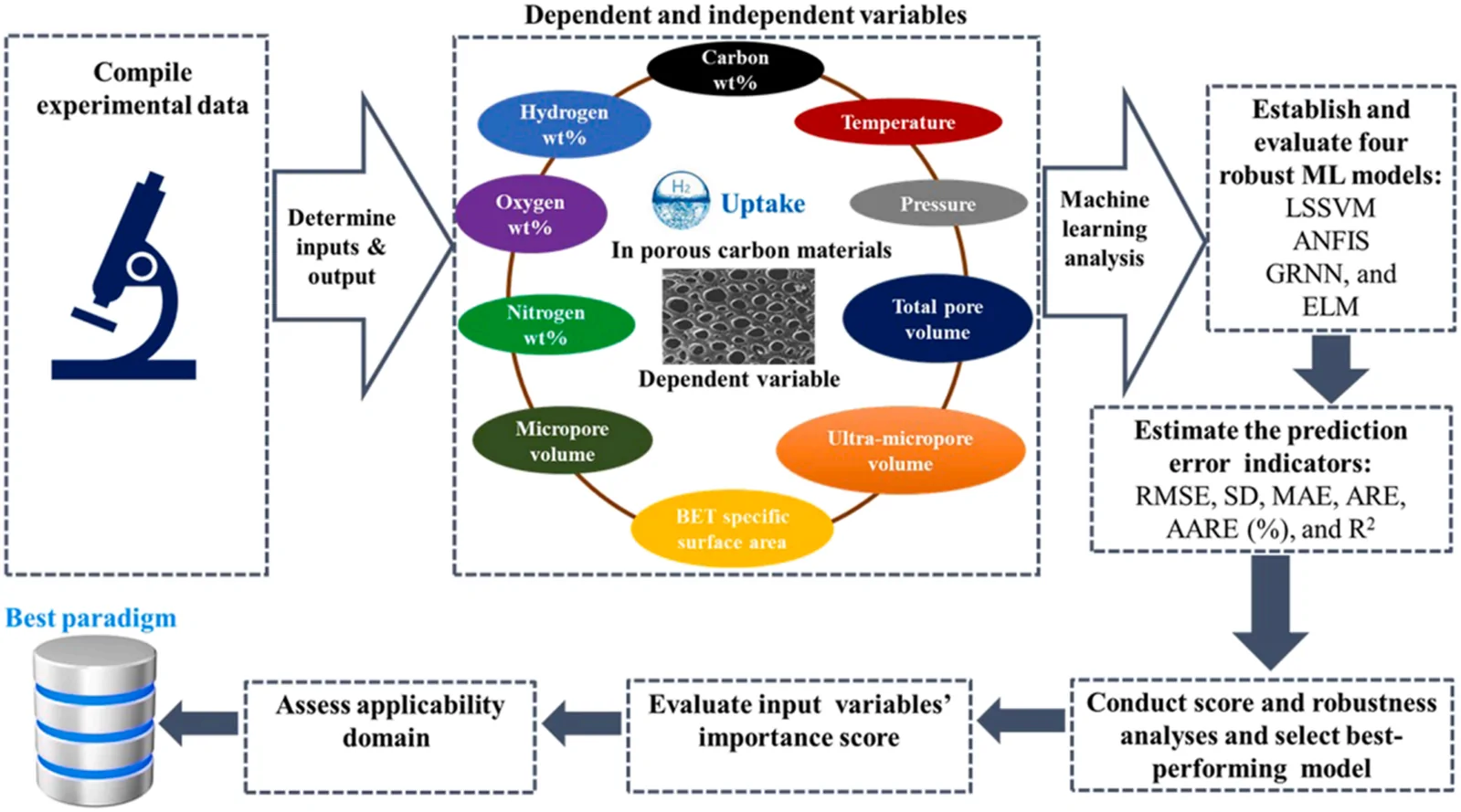
The use of machine learning can be effective in predicting hydrogen uptake values of porous carbon materials, using models like least-squares-support-vector machine (LSSVM), generalized-regression neural network (GRNN), extreme-learning machine (ELM), and adaptive-neuro-fuzzy-inference system (ANFIS). A database of 2072 records of hydrogen storage data is collected from the literature, including the BET surface area, ultrapore volume, hydrogen storage capacity, temperature, and pressure. This figure has been reproduced from ref. 298 with permission from Elsevier, copyright 2023.

Based on the large number of studies, the use of porous materials for hydrogen storage is promising. However, many challenges need to be addressed to integrate porous carbon and other porous materials into real world applications. As mentioned earlier, many challenges already exist for synthesis and material development. As highlighted by the US DOE, these materials should achieve the set goals of high gravimetric capacity, recyclability, and reasonable operating temperatures and pressure. However, not only are we facing challenges in the material development phase, but also in other steps along the material designing and manufacturing process including large-scale manufacturing and sustainability as highlighted under life cycle assessment. As we have seen from numerous reports, the discovery of materials for hydrogen storage is guided by experimental as well as theoretical predictions from computer simulations like DFT and GCMC. Despite their limitations,^286–289^ they have been proven to be helpful in understanding the interactions between carbon and hydrogen. Computational modelling coupled with other emerging prediction tools, such as machine learning, is being employed increasingly to improve the material selection and discovery process.^237,290^ Machine learning (ML) is a subset of artificial intelligence (AI) that uses statistical methods to process huge amounts of data and identify patterns that might not be visible using conventional methods. It can be categorized into supervised learning, unsupervised learning, semi-supervised learning and reinforcement learning.^291^ Recently, ML has been increasingly employed to aid in the process of porous material discovery and optimisation.^292^ In the context of porous materials for hydrogen storage, Giappa *et al.* have used a bottom up approach for predicting hydrogen storage capacity. *Ab initio* calculations of binding energy have been coupled with machine learning techniques to explore the effect of potential ligand functionalisation on hydrogen storage capacity of MOFs.^293^ Calculations have shown that the functional groups like –NH_2_ and –OPO_3_H_2_ enhance the H_2_ binding enthalpy by 16% and 26%, respectively, and –OSO_3_H_2_ functionalisation has predicted an enhancement of 81% when compared to the non-functionalised benzene ring. GCMC simulations were used under cryogenic and ambient conditions to simulate the hydrogen adsorption capacity of three IRMOFs using the strongest binding functional groups from *ab initio* calculations. In all cases, the results show an increased volumetric hydrogen uptake, which is in line with the increased binding energies from *ab initio* studies. Finally, ML was successfully used to predict binding energies using supervised machine learning. The study demonstrates the power of combining advanced computational methods with machine learning techniques to predict and optimize material properties in the field of hydrogen storage. In another study, GCMC was used as a validation technique for deep-learning models predicting deliverable hydrogen storage capacities in MOFs.^294^ Light Gradient Boosting Machine (LightGBM) and Random Forest (RF) ML models were employed using a database of 219 experimental samples of MOFs. In addition to a pre-processing of data down to 183 samples, the study has revealed that changes in H_2_ uptake were mostly affected by enthalpy of adsorption, followed by the surface area of MOFs.^295^ In another study, the random forest model and Shapley additive explanations (SHAP) were used on 68 samples and 1745 data points of activated carbon,^296^ and interesting structure–property relationships of activated carbon were revealed. It was found that the pressure and BET SSA are the most significant predictors of hydrogen uptake, while the pore volume had the smallest influence. Interestingly, oxygen content had a positive impact with enhanced uptake by about 0.6 wt%. Rahimi *et al.* investigated the optimization of properties of activated carbon for better hydrogen storage using machine learning techniques.^297^ The study has demonstrated the influence of micropore volume and pore widening on hydrogen storage. Davoodi *et al.* evaluated a database of 2072 records from 68 porous carbon samples, each with eleven identified dependent and independent variables using four ML learning models (as shown in Fig. 17).^298^ The authors have highlighted that machine learning can handle large database sets, learn and improve their predictions, and provide insight on hydrogen storage mechanisms in addition to identifying influential factors for enhancing hydrogen storage capacity. However, it can still suffer from disadvantages like requirement of expertise in data analysis and resources. Despite the challenges that are limited by its complexity and data quality,^299^ machine learning can enable rapid screening, prediction, and optimization of the material discovery process and can significantly reduce the time and cost associated with it.^300^ As more experimental and computational data become available through experimental and theoretical studies, contributing towards a larger database and more reliable data, the application of ML in this field is expected to grow as a validation and optimisation tool for material discovery, alongside experimental and computational techniques.

Another challenge in this field is identified as the wide discrepancy of practice among researchers, in measurement and reporting hydrogen uptake capacities. This particularly arises due to factors like variability in synthesis conditions, measurement errors, and lack of standard protocols in reporting data. For example, most available literature studies reporting hydrogen storage capacities correlate good uptake with a change in BET surface area. However, it was shown that the BET method has its own limitations when it comes to measuring the surface area of highly microporous materials.^301^ To overcome these problems, a series of recommendations by Broom and Hirscher, and others were suggested for the accurate measurement, calibration and avoidance of error. Suggestions have been made for reporting and presenting isotherm data, to ensure reproducibility and eliminate interlaboratory differences.^302,303^ Existing efforts for the standardisation and ease of access for adsorption data include the suggestion made by Evans *et al.* for establishing a decentralised adsorption database library for porous materials using proposed Adsorption Information File (AIF).^304^ Moreover, there is a need for reference materials with well-characterised and established synthesis and measurement protocols that can act as benchmark materials for calibration.^305^ These recommendations can also contribute towards the standardisation of reporting hydrogen storage capacities of various materials. As a routine practice, besides addressing the challenges faced in the material development phase, it would be also beneficial to consider lifecycle analysis, carbon balance and cost production analysis at the early phase of the material selection process, for a sustainable approach.^306^

## 6. Conclusion

Due to the challenges associated with physical properties of hydrogen, its efficient storage is one of the key barriers for successful implementation of hydrogen economy on a wider scale. Many physical storage methods have been introduced including compression and liquefaction. However, there is a growing interest in adsorption-based hydrogen storage in porous materials due to higher efficiency and low-pressure requirement. In this review, some of the key parameters for hydrogen storage in porous materials are discussed, including definitions of excess, net, and total as well as engineering and usable capacities. The effectiveness of hydrogen storage on different types of carbon has been discussed and it was observed that in addition to operating conditions (*e.g.*, pressure and temperature) the storage capacity can be affected by factors intrinsic to the materials, such as the surface area, pore size and surface chemistry, due to the presence of intermediary interactions like Kubas binding and the spillover effect. Porous carbon comes in the form of many different allotropes with unique morphologies and arrangements including graphene and nanotubes. They are versatile and attractive for hydrogen storage due to their high surface area and thermal and mechanical stability. In this review a wide range of porous carbon materials have been examined, ranging from graphene to activated carbon, concluding that in general carbon materials with higher surface areas and micropore volumes show high hydrogen storage capacities, with template-derived carbons and activated carbons showing the highest storage capacities reported to date. Besides the high surface area, various modifications and optimizations can further enhance hydrogen storage capacity, particularly using strategies that either (1) enhance the structural and morphological features of the material by introducing defects and/or increasing the surface area, or (2) incorporate functional groups, heteroatoms, or doped nanoparticles to make hydrogen adsorption kinetics under ambient conditions more favorable. Besides the synthetic strategies, the comparative overview of the performance of different classes of porous carbon along with the importance of LCA, and the reflection on the emerging trend of use of machine learning in this review can be useful for material selection and further improvement for developing superior porous carbons for hydrogen storage applications.

## Author contributions

Lila A. M. Mahmoud: writing – original draft. Jemma L. Rowlandson, David J. Fermin, Valeska P. Ting, and Sanjit Nayak: supervision; writing – review & editing.

## Conflicts of interest

There are no conflicts to declare.

## Data Availability

No primary research results, software or code have been included and no new data were generated or analysed as part of this review.

## References

[cit1] Meinshausen M., Meinshausen N., Hare W., Raper S. C. B., Frieler K., Knutti R., Frame D. J., Allen M. R. (2009). Nature.

[cit2] Shahbaz M., Raghutla C., Chittedi K. R., Jiao Z., Vo X. V. (2020). Energy.

[cit3] Winter C.-J. (2009). Int. J. Hydrogen Energy.

[cit4] Mazloomi K., Gomes C. (2012). Renewable Sustainable Energy Rev..

[cit5] Allendorf M. D., Stavila V., Snider J. L., Witman M., Bowden M. E., Brooks K., Tran B. L., Autrey T. (2022). Nat. Chem..

[cit6] Møller K. T., Jensen T. R., Akiba E., Li H.-w. (2017). Prog. Nat. Sci.: Mater. Int..

[cit7] Züttel A. (2003). Mater. Today.

[cit8] US. DOE , Target Explanation Document: Onboard Hydrogen Storage for Light-Duty Fuel Cell Vehicles, https://www.energy.gov/eere/fuelcells/downloads/target-explanation-document-onboard-hydrogen-storage-light-duty-fuel-cell, (accessed November 2024)

[cit9] Nazir G., Rehman A., Hussain S., Aftab S., Heo K., Ikram M., Patil S. A., Aizaz Ud Din M. (2022). Adv. Sustainable Syst..

[cit10] Züttel A. (2004). Sci. Nat..

[cit11] Zhang F., Zhao P., Niu M., Maddy J. (2016). Int. J. Hydrogen Energy.

[cit12] Folkson R. , in Alternative Fuels and Advanced Vehicle Technologies for Improved Environmental Performance, ed. R. Folkson and S. Sapsford, Woodhead Publishing, 2nd edn, 2022, pp. 151–171, 10.1016/B978-0-323-90979-2.00013-5

[cit13] Usman M. R. (2022). Renewable Sustainable Energy Rev..

[cit14] Al Ghafri S. Z. S., Munro S., Cardella U., Funke T., Notardonato W., Trusler J. P. M., Leachman J., Span R., Kamiya S., Pearce G., Swanger A., Rodriguez E. D., Bajada P., Jiao F., Peng K., Siahvashi A., Johns M. L., May E. F. (2022). Energy Environ. Sci..

[cit15] Cheng Q., Zhang R., Shi Z., Lin J. (2024). Int. J. Lightweight Mater. Manuf..

[cit16] Gil A. (2023). Catal. Today.

[cit17] Hirscher M., Panella B. (2005). J. Alloys Compd..

[cit18] Xia Y., Yang Z., Zhu Y. (2013). J. Mater. Chem. A.

[cit19] Hoang T. K. A., Antonelli D. M. (2009). Adv. Mater..

[cit20] ‘chemisorption’ in IUPAC Compendium of Chemical Terminology, International Union of Pure and Applied Chemistry, 3rd edn, 2006, Online version 3.0.1, 2019, 10.1351/goldbook.C01048

[cit21] Zhang Y., Wu S., Wang L., Zhang X. (2023). Frontiers in Energy.

[cit22] in Metal-Catalysed Reactions of Hydrocarbons, ed. G. C. Bond, Springer US, Boston, MA, 2005, pp. 93–152, 10.1007/0-387-26111-7_3

[cit23] Zaluska A., Zaluski L., Ström-Olsen J. O. (2000). J. Alloys Compd..

[cit24] Meduri S., Nandanavanam J. (2023). Mater. Today: Proc..

[cit25] Orimo S.-i., Nakamori Y., Eliseo J. R., Züttel A., Jensen C. M. (2007). Chem. Rev..

[cit26] Zhang X. L., Liu Y. F., Zhang X., Hu J. J., Gao M. X., Pan H. G. (2020). Mater. Today Nano.

[cit27] Khadilkar P., Samudre N. S., Krishnamurty S. (2024). J. Energy Storage.

[cit28] Chung C., Ihm J., Lee H. (2015). J. Korean Phys. Soc..

[cit29] Skipper C. V. J., Hamaed A., Antonelli D. M., Kaltsoyannis N. (2012). Dalton Trans..

[cit30] Sakintuna B., Lamari-Darkrim F., Hirscher M. (2007). Int. J. Hydrogen Energy.

[cit31] Broom D. P. , in Hydrogen Storage Materials: The Characterisation of Their Storage Properties, ed. D. P. Broom, Springer London, London, 2011, pp. 183–234, 10.1007/978-0-85729-221-6_6

[cit32] Han S. S., Furukawa H., Yaghi O. M., Goddard, III W. A. (2008). J. Am. Chem. Soc..

[cit33] Klontzas E., Tylianakis E., Froudakis G. E. (2010). Nano Lett..

[cit34] McKeown N. B., Budd P. M., Book D. (2007). Macromol. Rapid Commun..

[cit35] Ramimoghadam D., Gray E. M., Webb C. J. (2016). Int. J. Hydrogen Energy.

[cit36] Yin X., Alsuwaidi A., Zhang X. (2022). Microporous Mesoporous Mater..

[cit37] Farha O. K., Eryazici I., Jeong N. C., Hauser B. G., Wilmer C. E., Sarjeant A. A., Snurr R. Q., Nguyen S. T., Yazaydın A. Ö., Hupp J. T. (2012). J. Am. Chem. Soc..

[cit38] Lin J., Zhong Y., Tang L., Wang L., Yang M., Xia H. (2022). Nano Sel..

[cit39] Polak-Kraśna K., Dawson R., Holyfield L. T., Bowen C. R., Burrows A. D., Mays T. J. (2017). J. Mater. Sci..

[cit40] Ma X., Chen R., Zhou K., Wu Q., Li H., Zeng Z., Li L. (2020). ACS Sustainable Chem. Eng..

[cit41] Howarth A. J., Liu Y., Li P., Li Z., Wang T. C., Hupp J. T., Farha O. K. (2016). Nat. Rev. Mater..

[cit42] Côté A. P., Benin A. I., Ockwig N. W., O'Keeffe M., Matzger A. J., Yaghi O. M. (2005). Science.

[cit43] Budd P. M., Msayib K. J., Tattershall C. E., Ghanem B. S., Reynolds K. J., McKeown N. B., Fritsch D. (2005). J. Membr. Sci..

[cit44] Rochat S., Polak-Kraśna K., Tian M., Mays T. J., Bowen C. R., Burrows A. D. (2019). Int. J. Hydrogen Energy.

[cit45] Liu Z., Zang C., Ju Z., Hu D., Zhang Y., Jiang J., Liu C. (2020). J. Cleaner Prod..

[cit46] Zhang Q., Yan S., Yan X., Lv Y. (2023). Sci. Total Environ..

[cit47] McKeown N. B. (2017). Sci. China: Chem..

[cit48] Heidarinejad Z., Dehghani M. H., Heidari M., Javedan G., Ali I., Sillanpää M. (2020). Environ. Chem. Lett..

[cit49] Kobina Sam D., Li H., Xu Y.-T., Cao Y. (2024). J. Ind. Eng. Chem..

[cit50] Grünker R., Bon V., Müller P., Stoeck U., Krause S., Mueller U., Senkovska I., Kaskel S. (2014). Chem. Commun..

[cit51] Farha O. K., Özgür Yazaydın A., Eryazici I., Malliakas C. D., Hauser B. G., Kanatzidis M. G., Nguyen S. T., Snurr R. Q., Hupp J. T. (2010). Nat. Chem..

[cit52] Furukawa H., Yaghi O. M. (2009). J. Am. Chem. Soc..

[cit53] Blankenship L. S., Balahmar N., Mokaya R. (2017). Nat. Commun..

[cit54] Li Y., Guo Q., Ding Z., Jiang H., Yang H., Du W., Zheng Y., Huo K., Shaw L. L. (2024). Chem. Eng. J..

[cit55] Zou X. L., Zhou G., Duan W. H., Choi K., Ihm J. (2010). J. Phys. Chem. C.

[cit56] Ke Z. P., Cheng Y. Y., Yang S. Y., Li F., Ding L. F. (2017). Int. J. Hydrogen Energy.

[cit57] Du N., Robertson G. P., Song J., Pinnau I., Guiver M. D. (2009). Macromolecules.

[cit58] Satilmis B., Lanč M., Fuoco A., Rizzuto C., Tocci E., Bernardo P., Clarizia G., Esposito E., Monteleone M., Dendisová M., Friess K., Budd P. M., Jansen J. C. (2018). J. Membr. Sci..

[cit59] Han W., Zhang C., Zhao M., Yang F., Yang Y., Weng Y. (2021). J. Membr. Sci..

[cit60] Loh C. Y., Huang R., Bell R., Xie M. (2023). RSC Sustainability.

[cit61] DeSantis D., Mason J. A., James B. D., Houchins C., Long J. R., Veenstra M. (2017). Energy Fuels.

[cit62] Wang Y., Wang H., Liu Y., Peng M., Fan H., Meng H. (2024). Chin. Chem. Lett..

[cit63] Sing K. S. W. , in Studies in Surface Science and Catalysis, ed. F. Rodriguez-Reinoso, J. Rouquerol, K. S. W. Sing and K. K. Unger, Elsevier, 1991, vol. 62, pp. 1–9

[cit64] Nguyen C., Do D. D. (2001). Carbon.

[cit65] Shabir F., Sultan M., Miyazaki T., Saha B. B., Askalany A., Ali I., Zhou Y., Ahmad R., Shamshiri R. R. (2020). Renewable Sustainable Energy Rev..

[cit66] Ting V. P., Ramirez-Cuesta A. J., Bimbo N., Sharpe J. E., Noguera-Diaz A., Presser V., Rudic S., Mays T. J. (2015). ACS Nano.

[cit67] Gogotsi Y., Portet C., Osswald S., Simmons J. M., Yildirim T., Laudisio G., Fischer J. E. (2009). Int. J. Hydrogen Energy.

[cit68] Sethia G., Sayari A. (2016). Carbon.

[cit69] Zhang L., Allendorf M. D., Balderas-Xicohténcatl R., Broom D. P., Fanourgakis G. S., Froudakis G. E., Gennett T., Hurst K. E., Ling S., Milanese C., Parilla P. A., Pontiroli D., Riccò M., Shulda S., Stavila V., Steriotis T. A., Webb C. J., Witman M., Hirscher M. (2022). Prog. Energy.

[cit70] P. International Union of and C. Applied, 10.1351/goldbook.I03313

[cit71] Kloutse A. F., Zacharia R., Cossement D., Chahine R., Balderas-Xicohténcatl R., Oh H., Streppel B., Schlichtenmayer M., Hirscher M. (2015). Appl. Phys. A: Mater. Sci. Process..

[cit72] Nuhnen A., Janiak C. (2020). Dalton Trans..

[cit73] Purewal J. J., Kabbour H., Vajo J. J., Ahn C. C., Fultz B. (2009). Nanotechnology.

[cit74] Bhatia S. K., Myers A. L. (2006). Langmuir.

[cit75] Gao X., Zhong Z., Huang L., Mao Y., Wang H., Liu J., Ouyang L., Zhang L., Han M., Ma X., Zhu M. (2023). Nano Energy.

[cit76] Schaefer S., Jeder A., Sdanghi G., Gadonneix P., Abdedayem A., Izquierdo M. T., Maranzana G., Ouederni A., Celzard A., Fierro V. (2020). Int. J. Hydrogen Energy.

[cit77] Shen H., Li H., Yang Z., Li C. (2022). Green Energy Environ..

[cit78] Khoobiar S. (1964). J. Phys. Chem..

[cit79] Briggs N. M., Barrett L., Wegener E. C., Herrera L. V., Gomez L. A., Miller J. T., Crossley S. P. (2018). Nat. Commun..

[cit80] Psofogiannakis G. M., Froudakis G. E. (2009). J. Am. Chem. Soc..

[cit81] Singh P., Kulkarni M. V., Gokhale S. P., Chikkali S. H., Kulkarni C. V. (2012). Appl. Surf. Sci..

[cit82] Chen C.-H., Chung T.-Y., Shen C.-C., Yu M.-S., Tsao C.-S., Shi G.-N., Huang C.-C., Ger M.-D., Lee W.-L. (2013). Int. J. Hydrogen Energy.

[cit83] Jung H., Park K. T., Gueye M. N., So S. H., Park C. R. (2016). Int. J. Hydrogen Energy.

[cit84] Zacharia R., Rather S.-U., Hwang S. W., Nahm K. S. (2007). Chem. Phys. Lett..

[cit85] Sharpe J. E., Bimbo N., Ting V. P., Burrows A. D., Jiang D., Mays T. J. (2013). Adsorption.

[cit86] Minuto F. D., Balderas-Xicohténcatl R., Policicchio A., Hirscher M., Agostino R. G. (2018). Int. J. Hydrogen Energy.

[cit87] Myers A. L., Monson P. A. (2002). Langmuir.

[cit88] Parilla P. A., Gross K., Hurst K., Gennett T. (2016). Appl. Phys. A: Mater. Sci. Process..

[cit89] Gumma S., Talu O. (2010). Langmuir.

[cit90] Broom D. P. (2023). Adsorption.

[cit91] Murata K., El-Merraoui M., Kaneko K. (2001). J. Chem. Phys..

[cit92] Goldsmith J., Wong-Foy A. G., Cafarella M. J., Siegel D. J. (2013). Chem. Mater..

[cit93] Rochat S., Polak-Kraśna K., Tian M., Holyfield L. T., Mays T. J., Bowen C. R., Burrows A. D. (2017). J. Mater. Chem. A.

[cit94] Schmieder P., Grzywa M., Denysenko D., Hambach M., Volkmer D. (2015). Dalton Trans..

[cit95] Broom D. P., Webb C. J., Fanourgakis G. S., Froudakis G. E., Trikalitis P. N., Hirscher M. (2019). Int. J. Hydrogen Energy.

[cit96] Schlichtenmayer M., Hirscher M. (2016). Appl. Phys. A: Mater. Sci. Process..

[cit97] Thommes M., Kaneko K., Neimark A. V., Olivier J. P., Rodriguez-Reinoso F., Rouquerol J., Sing K. S. W. (2015). Pure Appl. Chem..

[cit98] Thomas K. M. (2007). Catal. Today.

[cit99] Amankwah K. A. G., Schwarz J. A. (1991). Int. J. Hydrogen Energy.

[cit100] Reza M. S., Yun C. S., Afroze S., Radenahmad N., Bakar M. S. A., Saidur R., Taweekun J., Azad A. K. (2020). Arab J. Basic Appl. Sci..

[cit101] Yang Z., Xia Y., Mokaya R. (2007). J. Am. Chem. Soc..

[cit102] Blankenship L. S., Mokaya R. (2017). Energy Environ. Sci..

[cit103] Baburin I. A., Klechikov A., Mercier G., Talyzin A., Seifert G. (2015). Int. J. Hydrogen Energy.

[cit104] Srinivas G., Zhu Y., Piner R., Skipper N., Ellerby M., Ruoff R. (2010). Carbon.

[cit105] Guo C. X., Wang Y., Li C. M. (2013). ACS Sustainable Chem. Eng..

[cit106] Zhou C. Y., Szpunar J. A. (2016). ACS Appl. Mater. Interfaces.

[cit107] Huang C.-C., Pu N.-W., Wang C.-A., Huang J.-C., Sung Y., Ger M.-D. (2011). Sep. Purif. Technol..

[cit108] Aboutalebi S. H., Aminorroaya-Yamini S., Nevirkovets I., Konstantinov K., Liu H. K. (2012). Adv. Energy Mater..

[cit109] Cho E. S., Ruminski A. M., Aloni S., Liu Y.-S., Guo J., Urban J. J. (2016). Nat. Commun..

[cit110] Jin C., Li J., Zhang K., Habibullah, Xia G., Wu C., Wang Y., Cen W., Chen Y., Yan Y., Chen Y. (2022). Nano Energy.

[cit111] Chen Y., Habibullah, Xia G., Jin C., Wang Y., Yan Y., Chen Y., Gong X., Lai Y., Wu C. (2023). Inorganics.

[cit112] Parambhath V. B., Nagar R., Ramaprabhu S. (2012). Langmuir.

[cit113] Ao Z. M., Peeters F. M. (2010). Phys. Rev. B.

[cit114] Chen Y. W., Habibullah, Xia G. H., Jin C. A., Wang Y., Yan Y. G., Chen Y. G., Gong X. F., Lai Y. Q., Wu C. L. (2023). Materials.

[cit115] Flamina A., Raghavendra R. M., Gupta A., Subramaniam A. (2023). Appl. Surf. Sci..

[cit116] Morandé A., Lillo P., Blanco E., Pazo C., Dongil A. B., Zarate X., Saavedra-Torres M., Schott E., Canales R., Videla A., Escalona N. (2023). J. Energy Storage.

[cit117] Zhao W., Fierro V., Zlotea C., Aylon E., Izquierdo M. T., Latroche M., Celzard A. (2011). Int. J. Hydrogen Energy.

[cit118] Sevilla M., Fuertes A. B., Mokaya R. (2011). Energy Environ. Sci..

[cit119] Jordá-Beneyto M., Lozano-Castelló D., Suárez-García F., Cazorla-Amorós D., Linares-Solano A. (2008). Microporous Mesoporous Mater..

[cit120] Sevilla M., Fuertes A. B., Mokaya R. (2011). Int. J. Hydrogen Energy.

[cit121] Ramirez-Vidal P., Canevesi R. L. S., Sdanghi G., Schaefer S., Maranzana G., Celzard A., Fierro V. (2021). ACS Appl. Mater. Interfaces.

[cit122] Sun Y., Webley P. A. (2010). Chem. Eng. J..

[cit123] Wang D., Geng Z., Zhang C., Zhou X., Liu X. (2014). J. Energy Chem..

[cit124] Ramesh T., Rajalakshmi N., Dhathathreyan K. S. (2015). J. Energy Storage.

[cit125] Minoda A., Oshima S., Iki H., Akiba E. (2013). J. Alloys Compd..

[cit126] Akasaka H., Takahata T., Toda I., Ono H., Ohshio S., Himeno S., Kokubu T., Saitoh H. (2011). Int. J. Hydrogen Energy.

[cit127] Li Y., Yang R. T. (2007). J. Phys. Chem. C.

[cit128] Pedicini R., Maisano S., Chiodo V., Conte G., Policicchio A., Agostino R. G. (2020). Int. J. Hydrogen Energy.

[cit129] Zhang J., Gao J., Chen Y., Hao X., Jin X. (2017). Results Phys..

[cit130] Bader N., Ouederni A. (2017). J. Energy Storage.

[cit131] Turkyilmaz A., Isinkaralar K., Dogan M., Kizilduman B. K., Bicil Z. (2024). Sustainable Chem. Pharm..

[cit132] Flamina A., Raghavendra R. M., Subramaniam A. (2024). Carbon.

[cit133] Peng Z., Xu Y., Luo W., Wang C., Ma L. (2020). ChemistrySelect.

[cit134] Li Y., Yang R. T., Liu C.-J., Wang Z. (2007). Ind. Eng. Chem. Res..

[cit135] Geng Z., Wang D., Zhang C., Zhou X., Xin H., Liu X., Cai M. (2014). Int. J. Hydrogen Energy.

[cit136] Figueroa-Torres M. Z., Domínguez-Ríos C., Cabañas-Moreno J. G., Vega-Becerra O., Aguilar-Elguézabal A. (2012). Int. J. Hydrogen Energy.

[cit137] Ruz P., Banerjee S., Das T., Kumar A., Sudarsan V., Patra A. K., Sastry P. U. (2024). J. Porous Mater..

[cit138] Adeniran B., Mokaya R. (2015). Nano Energy.

[cit139] Muhammad R., Nah Y. C., Oh H. (2023). J. CO2 Util.

[cit140] Li X., Tian H., Yan S., Shi H. J., Wu J. B., Sun Y. L., Xing Y. Q., Bai H. C., Zhang H. (2024). Int. J. Hydrogen Energy.

[cit141] Balathanigaimani M. S., Haider M. B., Jha D., Kumar R., Lee S. J., Shim W. G., Shon H. K., Kim S. C., Moon H. (2018). J. Nanosci. Nanotechnol..

[cit142] Jin H., Lee Y. S., Hong I. (2007). Catal. Today.

[cit143] Musyoka N. M., Mutuma B. K., Manyala N. (2020). RSC Adv..

[cit144] Chen T., Zhou Y., Luo L., Wu X., Li Z., Fan M., Zhao W. (2019). Electrochim. Acta.

[cit145] Serafin J., Dziejarski B., Solis C., Ramírez de la Piscina P., Homs N. (2024). Fuel.

[cit146] Lionetti V., Poselle Bonaventura C., Conte G., De Luca O., Policicchio A., Caruso T., Desiderio G., Papagno M., Agostino R. G. (2024). Int. J. Hydrogen Energy.

[cit147] Çetingürbüz E., Turkyilmaz A. (2023). Ind. Crops Prod..

[cit148] Segakweng T., Musyoka N. M., Ren J. W., Crouse P., Langmi H. W. (2016). Res. Chem. Intermed..

[cit149] Stadie N. P., Vajo J. J., Cumberland R. W., Wilson A. A., Ahn C. C., Fultz B. (2012). Langmuir.

[cit150] Almasoudi A., Mokaya R. (2012). J. Mater. Chem..

[cit151] Xia Y., Yang Z., Mokaya R. (2010). Chem. Vap. Deposition.

[cit152] Campesi R., Cuevas F., Gadiou R., Leroy E., Hirscher M., Vix-Guterl C., Latroche M. (2008). Carbon.

[cit153] Almasoudi A., Mokaya R. (2014). J. Mater. Chem. A.

[cit154] Juárez J. M., Gómez M. B., Anunziata O. A. (2015). Int. J. Energy Res..

[cit155] Cai J., Yang M., Xing Y., Zhao X. (2014). Colloids Surf., A.

[cit156] Kapsi M., Veziri C. M., Pilatos G., Karanikolos G. N., Romanos G. E. (2022). Int. J. Hydrogen Energy.

[cit157] Konwar R. J., De M. (2013). Microporous Mesoporous Mater..

[cit158] Masika E., Mokaya R. (2013). Prog. Nat. Sci.: Mater. Int..

[cit159] Xia Y., Mokaya R., Grant D. M., Walker G. S. (2011). Carbon.

[cit160] Attia N. F., Lee S. M., Kim H. J., Geckeler K. E. (2013). Microporous Mesoporous Mater..

[cit161] Alam N., Mokaya R. (2011). Microporous Mesoporous Mater..

[cit162] Alam N., Mokaya R. (2011). Microporous Mesoporous Mater..

[cit163] Konwar R. J., De M. (2014). J. Anal. Appl. Pyrolysis.

[cit164] Tseng P.-S., Chang L.-X., Ou Y.-S., Chou C.-M., Tsao C.-S., Wu Y., Chou J.-P., Chen P.-J., Wang C.-Y. (2023). Appl. Surf. Sci..

[cit165] Yang Z., Jia Q., Chen B., Gou X., Zhu Y., Xia Y. (2020). Int. J. Hydrogen Energy.

[cit166] Mosquera-Vargas E., Tamayo R., Morel M., Roble M., Díaz-Droguett D. E. (2021). Heliyon.

[cit167] Khalilov U., Uljayev U., Mehmonov K., Nematollahi P., Yusupov M., Neyts E. (2024). Int. J. Hydrogen Energy.

[cit168] Brewster C. D., Terry L. R., Doan H. V., Rochat S., Ting V. P. (2023). Energy Adv..

[cit169] Konni M., Dadhich A. S., Babu Mukkamala S. (2017). Int. J. Hydrogen Energy.

[cit170] Rakhi R. B., Sethupathi K., Ramaprabhu S. (2008). Int. J. Hydrogen Energy.

[cit171] Çalışır Ü., Çiçek B., Doğan M. (2021). ullerenes, Nanotubes Carbon Nanostruct..

[cit172] Rashidi A. M., Nouralishahi A., Khodadadi A. A., Mortazavi Y., Karimi A., Kashefi K. (2010). Int. J. Hydrogen Energy.

[cit173] Lee J. H., Rhee K. Y., Park S. J. (2010). Int. J. Hydrogen Energy.

[cit174] Vellingiri L., Annamalai K., Kandasamy R., Kombiah I. (2018). Int. J. Hydrogen Energy.

[cit175] Larijani M. M., Safa S. (2014). Acta Phys. Pol., A.

[cit176] Rather S.-u., Nahm K. S. (2014). Mater. Res. Bull..

[cit177] Lee S.-Y., Park S.-J. (2012). J. Solid State Chem..

[cit178] Kajiura H., Tsutsui S., Kadono K., Kakuta M., Ata M., Murakami Y. (2003). Appl. Phys. Lett..

[cit179] Sawant S. V., Banerjee S., Patwardhan A. W., Joshi J. B., Dasgupta K. (2020). Int. J. Hydrogen Energy.

[cit180] Aghababaei M., Ghoreyshi A. A., Esfandiari K. (2020). Int. J. Hydrogen Energy.

[cit181] Sharma A. (2020). Int. J. Hydrogen Energy.

[cit182] Rajaura R. S., Srivastava S., Sharma P. K., Mathur S., Shrivastava R., Sharma S. S., Vijay Y. K. (2018). Nano-Struct. Nano-Objects.

[cit183] Çakır Ü., Kestel F., Kızılduman B. K., Bicil Z., Doğan M. (2021). Diamond Relat. Mater..

[cit184] Kaskun S., Kayfeci M. (2018). Int. J. Hydrogen Energy.

[cit185] Barghi S. H., Tsotsis T. T., Sahimi M. (2014). Int. J. Hydrogen Energy.

[cit186] Silambarasan D., Surya V. J., Iyakutti K., Asokan K., Vasu V., Kawazoe Y. (2017). Appl. Surf. Sci..

[cit187] Dwivedi S. (2022). Int. J. Hydrogen Energy.

[cit188] Choi Y. S. , Yoo J. M. and Hong B. H., in Graphene for Flexible Lighting and Displays, ed. T.-W. Lee, Woodhead Publishing, 2020, pp. 5–26, 10.1016/B978-0-08-102482-9.00002-2

[cit189] Tiwari S. K., Sahoo S., Wang N., Huczko A. (2020). J. Sci.: Adv. Mater. Devices.

[cit190] Peigney A., Laurent C., Flahaut E., Bacsa R. R., Rousset A. (2001). Carbon.

[cit191] Okamoto Y., Miyamoto Y. (2001). J. Phys. Chem. B.

[cit192] Costanzo F., Silvestrelli P. L., Ancilotto F. (2012). J. Chem. Theory Comput..

[cit193] Patchkovskii S., Tse J. S., Yurchenko S. N., Zhechkov L., Heine T., Seifert G. (2005). Proc. Natl. Acad. Sci. U. S. A..

[cit194] Morse J. R., Zugell D. A., Patterson E., Baldwin J. W., Willauer H. D. (2021). J. Energy Storage.

[cit195] Kaczmarek L., Warga T., Zawadzki P., Makowicz M., Bucholc B., Kula P. (2019). Int. J. Hydrogen Energy.

[cit196] Subrahmanyam K. S., Kumar P., Maitra U., Govindaraj A., Hembram K. P. S. S., Waghmare U. V., Rao C. N. R. (2011). Proc. Natl. Acad. Sci. U. S. A..

[cit197] Alekseeva O. K., Pushkareva I. V., Pushkarev A. S., Fateev V. N. (2020). Nanotechnol. Russ..

[cit198] Ghosh A., Subrahmanyam K. S., Krishna K. S., Datta S., Govindaraj A., Pati S. K., Rao C. N. R. (2008). J. Phys. Chem. C.

[cit199] Lopez-Corral I., German E., Brizuela G. P., Juan A., Volpe M. A. (2010). Int. J. Hydrogen Energy.

[cit200] Cheng H., Chen L., Cooper A. C., Sha X., Pez G. P. (2008). Energy Environ. Sci..

[cit201] Jastrzębski K., Kula P. (2021). Materials.

[cit202] Long J., Li J. Y., Nan F., Yin S., Li J. J., Cen W. L. (2018). Appl. Surf. Sci..

[cit203] Ghotia S., Rimza T., Singh S., Dwivedi N., Srivastava A. K., Kumar P. (2024). J. Mater. Chem. A.

[cit204] Denis P. A. (2022). ACS Omega.

[cit205] Lee S. J., Theerthagiri J., Nithyadharseni P., Arunachalam P., Balaji D., Madan Kumar A., Madhavan J., Mittal V., Choi M. Y. (2021). Renewable Sustainable Energy Rev..

[cit206] Vinayan B. P., Nagar R., Ramaprabhu S. (2013). J. Mater. Chem. A.

[cit207] Anikina E., Naqvi S. R., Bae H., Lee H., Luo W., Ahuja R., Hussain T. (2022). Int. J. Hydrogen Energy.

[cit208] Cho Y., Kang S., Wood B. C., Cho E. S. (2022). ACS Appl. Mater. Interfaces.

[cit209] Tarasov B. P., Arbuzov A. A., Volodin A. A., Fursikov P. V., Mozhzhuhin S. A., Lototskyy M. V., Yartys V. A. (2022). J. Alloys Compd..

[cit210] Zhang W., Xu G., Chen L., Pan S., Jing X., Wang J., Han S. (2017). Int. J. Hydrogen Energy.

[cit211] Ruse E., Buzaglo M., Pri-Bar I., Shunak L., Nadiv R., Pevzner S., Siton-Mendelson O., Skripnyuk V. M., Rabkin E., Regev O. (2018). Carbon.

[cit212] Klechikov A., Mercier G., Sharifi T., Baburin I. A., Seifert G., Talyzin A. V. (2015). Chem. Commun..

[cit213] Liu Y., Zhang Z., Wang T. (2018). Int. J. Hydrogen Energy.

[cit214] Peng D. D., Ding Z. M., Zhang L., Fu Y. K., Wang J. S., Li Y., Han S. M. (2018). Int. J. Hydrogen Energy.

[cit215] Züttel A., Orimo S.-I. (2002). MRS Bull..

[cit216] Dillon A. C., Jones K. M., Bekkedahl T. A., Kiang C. H., Bethune D. S., Heben M. J. (1997). Nature.

[cit217] Liu C., Chen Y., Wu C.-Z., Xu S.-T., Cheng H.-M. (2010). Carbon.

[cit218] Lyu J., Kudiiarov V., Lider A. (2020). Nanomaterials.

[cit219] Froudakis G. E. (2011). Mater. Today.

[cit220] Chen C.-H., Huang C.-C. (2007). Int. J. Hydrogen Energy.

[cit221] Liu F., Zhang X., Cheng J., Tu J., Kong F., Huang W., Chen C. (2003). Carbon.

[cit222] Reyhani A., Mortazavi S. Z., Mirershadi S., Moshfegh A. Z., Parvin P., Golikand A. N. (2011). J. Phys. Chem. C.

[cit223] Liu W., Zhao Y. H., Li Y., Jiang Q., Lavernia E. J. (2009). J. Phys. Chem. C.

[cit224] Silambarasan D., Surya V. J., Iyakutti K., Vasu V. (2014). Int. J. Hydrogen Energy.

[cit225] Attia N. F., Menemparabath M. M., Arepalli S., Geckeler K. E. (2013). Int. J. Hydrogen Energy.

[cit226] Silambarasan D., Vasu V., Iyakutti K. (2014). IEEE Trans. Nanotechnol..

[cit227] Cheng H., Cooper A. C., Pez G. P., Kostov M. K., Piotrowski P., Stuart S. J. (2005). J. Phys. Chem. B.

[cit228] Cheng H.-M., Yang Q.-H., Liu C. (2001). Carbon.

[cit229] Jordá-Beneyto M., Suárez-García F., Lozano-Castelló D., Cazorla-Amorós D., Linares-Solano A. (2007). Carbon.

[cit230] Mohan M., Sharma V. K., Kumar E. A., Gayathri V. (2019). Energy Storage.

[cit231] Zhou L. (2006). Int. J. Hydrogen Energy.

[cit232] Kostoglou N., Koczwara C., Stock S., Tampaxis C., Charalambopoulou G., Steriotis T., Paris O., Rebholz C., Mitterer C. (2022). Chem. Eng. J..

[cit233] Deng J., Xiong T., Wang H., Zheng A., Wang Y. (2016). ACS Sustainable Chem. Eng..

[cit234] Syed-Hassan S. S. A., Zaini M. S. M. (2016). Korean J. Chem. Eng..

[cit235] Sankir M. and Sankir N. D., Hydrogen Storage Technologies, John Wiley & Sons, Incorporated, Newark, United States, 2018

[cit236] Lua A. C. (2020). Biomass Convers. Biorefin..

[cit237] Rowlandson J. L., Coombs OBrien J., Edler K. J., Tian M., Ting V. P. (2019). C.

[cit238] Zhao C., Ge L., Li X., Zuo M., Xu C., Chen S., Li Q., Wang Y., Xu C. (2023). Energy.

[cit239] Zhao W. G., Luo L., Wang H. Y., Fan M. Z. (2017). BioResources.

[cit240] Wang J., Senkovska I., Kaskel S., Liu Q. (2014). Carbon.

[cit241] Morande A., Lillo P., Blanco E., Pazo C., Dongil A. B., Zarate X., Saavedra-Torres M., Schott E., Canales R., Videla A., Escalona N. (2023). J. Energy Storage.

[cit242] Hu J., Gao Q., Wu Y., Song S. (2007). Int. J. Hydrogen Energy.

[cit243] Hossain M. S., Furusawa T., Sato M. (2023). Inorg. Chem. Commun..

[cit244] Jiang H.-L., Liu B., Lan Y.-Q., Kuratani K., Akita T., Shioyama H., Zong F., Xu Q. (2011). J. Am. Chem. Soc..

[cit245] Chaikittisilp W., Ariga K., Yamauchi Y. (2013). J. Mater. Chem. A.

[cit246] Xia Y. D., Yang Z. X., Gou X. L., Zhu Y. Q. (2013). Int. J. Hydrogen Energy.

[cit247] Batten S. R., Champness N. R., Chen X.-M., Garcia-Martinez J., Kitagawa S., Öhrström L., O'Keeffe M., Suh M. P., Reedijk J. (2013). Pure Appl. Chem..

[cit248] Yang S. J., Kim T., Im J. H., Kim Y. S., Lee K., Jung H., Park C. R. (2012). Chem. Mater..

[cit249] Sdanghi G. , Sdanghi G., Maranzana G., Celzard A. and Fierro V., in Hydrogen Storage Technologies, 2018, ch. 9, pp. 263–320, 10.1002/9781119460572

[cit250] Sevilla M., Foulston R., Mokaya R. (2010). Energy Environ. Sci..

[cit251] Kobielska P. A., Telford R., Rowlandson J., Tian M., Shahin Z., Demessence A., Ting V. P., Nayak S. (2018). ACS Appl. Mater. Interfaces.

[cit252] Yushin G., Dash R., Jagiello J., Fischer J. E., Gogotsi Y. (2006). Adv. Funct. Mater..

[cit253] Baca M., Cendrowski K., Kukulka W., Bazarko G., Moszyński D., Michalkiewicz B., Kalenczuk R. J., Zielinska B. (2018). Nanomaterials.

[cit254] Li Y., Li D., Rao Y., Zhao X., Wu M. (2016). Carbon.

[cit255] Islam A.-A. G. A., Janaun J., Mastuli M. S., Taufiq-Yap Y. H. (2016). Mater. Lett..

[cit256] Chambers A., Park C., Baker R. T. K., Rodriguez N. M. (1998). J. Phys. Chem. B.

[cit257] Jin P. and Gu X., in Handbook of Fullerene Science and Technology, ed. X. Lu, T. Akasaka and Z. Slanina, Springer Singapore, Singapore, 2021, pp. 1–30, 10.1007/978-981-13-3242-5_22-1

[cit258] Margadonna S. and Prassides K., in Encyclopedia of Materials: Science and Technology, ed. K. H. J. Buschow, R. W. Cahn, M. C. Flemings, B. Ilschner, E. J. Kramer, S. Mahajan and P. Veyssière, Elsevier, Oxford, 2001, pp. 3379–3383, 10.1016/B0-08-043152-6/00603-3

[cit259] Zhao Y., Kim Y.-H., Dillon A. C., Heben M. J., Zhang S. B. (2005). Phys. Rev. Lett..

[cit260] Yildirim T., Íñiguez J., Ciraci S. (2005). Phys. Rev. B.

[cit261] Muralikrishna I. V. and Manickam V., in Environmental Management, ed. I. V. Muralikrishna and V. Manickam, Butterworth-Heinemann, 2017, pp. 57–75, 10.1016/B978-0-12-811989-1.00005-1

[cit262] Temizel-Sekeryan S., Wu F., Hicks A. L. (2021). Int. J. Life Cycle Assess..

[cit263] Mubarak N. M., Abdullah E. C., Jayakumar N. S., Sahu J. N. (2014). J. Ind. Eng. Chem..

[cit264] Teah H. Y., Sato T., Namiki K., Asaka M., Feng K., Noda S. (2020). ACS Sustainable Chem. Eng..

[cit265] Isaacs J. A., Tanwani A., Healy M. L., Dahlben L. J. (2010). J. Nanopart. Res..

[cit266] Upadhyayula V. K. K., Meyer D. E., Curran M. A., Gonzalez M. A. (2012). J. Cleaner Prod..

[cit267] Wernet G., Bauer C., Steubing B., Reinhard J., Moreno-Ruiz E., Weidema B. (2016). Int. J. Life Cycle Assess..

[cit268] Healy M. L., Dahlben L. J., Isaacs J. A. (2008). J. Ind. Ecol..

[cit269] Gavankar S., Suh S., Keller A. A. (2015). J. Ind. Ecol..

[cit270] He K., Zhang Z.-Y., Zhang F.-S. (2021). Waste Manage..

[cit271] Jia C., Pang M., Lu Y., Liu Y., Zhuang M., Liu B., Lu J., Wei T., Wang L., Bian T., Wang M., Yu F., Sun L., Lin L., Teng T., Wu X., He Z., Gao J., Luo J., Zhang S., Feng L., Yin X., You F., Li G., Zhang L., Zhu Y.-G., Zhu X., Yang Y. (2022). One Earth.

[cit272] Arena N., Lee J., Clift R. (2016). J. Cleaner Prod..

[cit273] Vilén A., Laurell P., Vahala R. (2022). J. Environ. Manage..

[cit274] Wang Y., Wang J., Zhang X., Bhattacharyya D., Sabolsky E. M. (2022). Energies.

[cit275] Li C., Sun K., Sun Y., Shao Y., Gao G., Zhang L., Zhang S., Hu X. (2024). Chem. Eng. J..

[cit276] Hischier R., Walser T. (2012). Sci. Total Environ..

[cit277] Hassan Q., Algburi S., Sameen A. Z., Salman H. M., Jaszczur M. (2024). Int. J. Hydrogen Energy.

[cit278] Razmi A. R., Hanifi A. R., Shahbakhti M. (2023). Renewable Energy.

[cit279] Esposito L., van der Wiel M., Acar C. (2024). Int. J. Hydrogen Energy.

[cit280] Muthukumar P., Kumar A., Afzal M., Bhogilla S., Sharma P., Parida A., Jana S., Kumar E. A., Pai R. K., Jain I. P. (2023). Int. J. Hydrogen Energy.

[cit281] Ghorbani Y., Zhang S. E., Nwaila G. T., Bourdeau J. E., Rose D. H. (2024). J. Cleaner Prod..

[cit282] Younas M., Shafique S., Hafeez A., Javed F., Rehman F. (2022). Fuel.

[cit283] Di Micco S., Romano F., Jannelli E., Perna A., Minutillo M. (2023). Int. J. Hydrogen Energy.

[cit284] Caliskan H., Dincer I., Hepbasli A. (2013). Appl. Therm. Eng..

[cit285] Khosravi A., Koury R. N. N., Machado L., Pabon J. J. G. (2018). Energy.

[cit286] Broom D. P., Webb C. J., Hurst K. E., Parilla P. A., Gennett T., Brown C. M., Zacharia R., Tylianakis E., Klontzas E., Froudakis G. E., Steriotis T. A., Trikalitis P. N., Anton D. L., Hardy B., Tamburello D., Corgnale C., van Hassel B. A., Cossement D., Chahine R., Hirscher M. (2016). Appl. Phys. A: Mater. Sci. Process..

[cit287] Caviedes D., Cabria I. (2022). Int. J. Hydrogen Energy.

[cit288] Thomas K. M. (2009). Dalton Trans..

[cit289] Wu X. J., Wang Y. G., Cai Z. J., Zhao D. M., Cai W. Q. (2020). Chem. Eng. Sci..

[cit290] Thanh H. V., Ebrahimnia Taremsari S., Ranjbar B., Mashhadimoslem H., Rahimi E., Rahimi M., Elkamel A. (2023). Energies.

[cit291] Osman A. I., Abd-Elaziem W., Nasr M., Farghali M., Rashwan A. K., Hamada A., Wang Y. M., Darwish M. A., Sebaey T. A., Khatab A., Elsheikh A. H. (2024). Environ. Chem. Lett..

[cit292] Salehi K., Rahmani M., Atashrouz S. (2023). Int. J. Hydrogen Energy.

[cit293] Giappa R. M., Tylianakis E., Di Gennaro M., Gkagkas K., Froudakis G. E. (2021). Int. J. Hydrogen Energy.

[cit294] Shekhar S., Chowdhury C. (2024). Surf. Interfaces.

[cit295] Seyyedattar M., Zendehboudi S., Ghamartale A., Afshar M. (2024). Int. J. Hydrogen Energy.

[cit296] Maulana Kusdhany M. I., Lyth S. M. (2021). Carbon.

[cit297] Rahimi M., Abbaspour-Fard M. H., Rohani A. (2021). J. Cleaner Prod..

[cit298] Davoodi S., Vo Thanh H., Wood D. A., Mehrad M., Al-Shargabi M., Rukavishnikov V. S. (2023). Sep. Purif. Technol..

[cit299] Osman A. I., Nasr M., Farghali M., Rashwan A. K., Abdelkader A., Al-Muhtaseb A. A. H., Ihara I., Rooney D. W. (2024). Environ. Chem. Lett..

[cit300] Mobarak M. H., Mimona M. A., Islam M. A., Hossain N., Zohura F. T., Imtiaz I., Rimon M. I. H. (2023). Appl. Surf. Sci..

[cit301] Schlapbach L., Züttel A. (2001). Nature.

[cit302] Zlotea C., Moretto P., Steriotis T. (2009). Int. J. Hydrogen Energy.

[cit303] Broom D. P., Hirscher M. (2021). ChemPhysChem.

[cit304] Evans J. D., Bon V., Senkovska I., Kaskel S. (2021). Langmuir.

[cit305] Hruzewicz-Kołodziejczyk A., Ting V. P., Bimbo N., Mays T. J. (2012). Int. J. Hydrogen Energy.

[cit306] Kahilu G. M., Bada S., Mulopo J. (2023). Waste Dispos. Sustain. Energy..

